# Experimental and Monte Carlo Insights into the Biofilm Control of *Klebsiella pneumoniae* Using Green-Synthesized Ag/Ni(OH)_2_/Clove Nanocomposites

**DOI:** 10.3390/nano16191202

**Published:** 2026-09-23

**Authors:** Sahar Abdel Aleem Abdel Aziz, Samar M. Mahgoub, Mohamed M. Abdel Rahim, Eman A. Mohamed, Haifa E. Alfassam, Fatma Mohammed Ahmed, Saleh Maoda, Sherin R. Rouby, Ghada S. I. Osman, Afaf Alatawi, Rehab Mahmoud

**Affiliations:** 1 Hygiene, Zoonoses and Epidemiology Department, Faculty of Veterinary Medicine, Beni Suef University, Beni Suef 62511, Egypt; abdelaziz.sahar@yahoo.com (S.A.A.A.A.);; 2 Materials Science and Nanotechnology Department, Faculty of Postgraduate Studies for Advanced Sciences, Beni Suef University, Beni Suef 62511, Egypt; 3 Renewable Energy Science and Engineering Department, Faculty of Postgraduate Studies for Advanced Sciences (PSAS), Beni Suef University, Beni Suef 62511, Egypt; 4 Biology Department, College of Science, Princess Nourah Bint Abdulrahman University, Riyadh 11671, Saudi Arabia; 5 Microbiology and Biochemistry Department, Faculty of Science, Beni Suef University, Beni Suef 62511, Egypt; 6 Department of Zoology, College of Science, King Saud University, P.O. Box 2455, Riyadh 11451, Saudi Arabia; 7 Veterinary Medicine Department, Faculty of Veterinary Medicine, Beni Suef University, Beni Suef 62511, Egypt; 8 Bacteriology Department, Animal Health Research Institute (AHRI), Agricultural Research Center (ARC), Beni Suef 62511, Egypt; 9 Biology Department, Collage of Science, Tabuk University, Tabuk 71491, Saudi Arabia; afalatawi@ut.edu.sa; 10 Chemistry Department, Faculty of Science, Beni Suef University, Beni Suef 62511, Egypt

**Keywords:** *Klebsiella pneumoniae*, resistance, biofilm, cattle, stool, green chemistry, safety

## Abstract

One of the most significant challenges with disease control in humans and animals is biofilm formation by *Klebsiella pneumoniae* (*K. pneumoniae*). The current study investigated the prevalence of *K. pneumoniae* in fecal and milk samples from cattle and humans, along with their antimicrobial susceptibility patterns and biofilm formation, using conventional methods. In addition, the efficacy of Ag/Ni(OH)_2_/Clove and Ag/Ni(OH)_2_/C nanocomposites was comparatively evaluated to combat resistance and biofilm-associated traits. *K. pneumoniae* was recovered from 48.0%, 38.0% and 43.3% of the fecal, milk and stool samples, respectively. Moreover, antimicrobial susceptibility testing revealed a noteworthy pattern shared between human and animal samples. Furthermore, a Congo Red (CRA) assay and molecular detection of the biofilm-associated genes iut-A and mrk-A demonstrated that the majority of *K. pneumoniae* isolates exhibited biofilm characteristics. Using a green chemistry approach, Ag/Ni nanoparticles displayed greater bactericidal activity in the calcined form, as evidenced by a lower MIC (20.83 ± 5.23 μg/mL), an increase in the inhibition zone diameter (20.67 ± 1.76 mm), and low cytotoxicity toward HEK293 cells that was evident only within the lower tested concentration range (7.8–62.5 µg/mL), where cell viability remained above 90.0%; viability declined markedly at higher concentrations, with IC50 values of 264.7 µg/mL for Ag/Ni(OH)2/Clove and 282.8 µg/mL for Ag/Ni(OH)2/C. Notably, compared with Ag/Ni(OH)_2_/Clove, Ag/Ni(OH)_2_/C nanocomposites exhibited stronger antibiofilm properties. Likewise, the green synthesis of Ag/Ni(OH)_2_/Clove and Ag/Ni(OH)_2_/C nanocomposites represents an efficient approach for improving safety profiles in biological applications when applied within this lower, biocompatible concentration range, particularly against microbial strains exhibiting drug resistance and biofilm formation. Monte Carlo (MC) simulations were performed to investigate the interactions of the Ag/Ni(OH)2 composite with lipopolysaccharide (LPS), ergosterol, and chitin, which represent bacterial and fungal cell wall components. The negative adsorption energies (−50.15, −23.46, and −8.08 kcal/mol) indicated strong adsorption, supporting the experimentally observed antibacterial and antifungal activities of Ag/Ni(OH)_2_ composite.

## 1. Introduction

Biofilm infections remain significant issues across the medical field. Both Gram-negative bacteria and Gram-positive bacteria may develop biofilms, as exemplified by *Klebsiella pneumoniae* (*K. pneumoniae*), which are linked to elevated morbidity rates, substantial financial losses, and the potential for chronic infection [1]. Furthermore, the primary virulence determinants of *K. pneumoniae* include its ability to overgrow on biological and/or abiotic surface areas, as well as biofilm formation, in which the bacteria are encased in an extracellular polymeric matrix composed of polysaccharides, proteins, and DNA. Moreover, the formation of biofilms by *Klebsiella* species shields microorganisms from phagocytosis, antibody activity, and expulsion by pathogens through the ciliary action of epithelial cells. Consequently, this increases the resistance of *Klebsiella* spp. to the host immune system and antimicrobial agents [2].

*Klebsiella* species are encapsulated Gram-negative, facultative anaerobic bacilli that belong to the *Enterobacteriaceae* family [3]. These bacteria are ubiquitous in the environment and are part of the normal flora in human and animal gastrointestinal tracts [4]. Moreover, *K. pneumoniae* is an opportunistic pathogen that can cause several life-threatening nosocomial-associated infections, including septicemia, meningitis, pneumonia, soft tissue infections, urinary tract infections, and pyogenic liver abscesses in mammals. They are also the main causes of mastitis, pneumonia, and metritis in cattle [5]. Multidrug-resistant (MDR) or antimicrobial-resistant (AR) bacterial pathogens cause numerous severe human infections and are considered among the three main threats facing the world. *K. pneumoniae* is considered the second-deadliest Gram-negative bacterium that causes both hospital- and community-acquired infections and has witnessed an abrupt increase in antibiotic resistance [6].

The use of novel therapeutic approaches based on nanotechnology, including metal-based nanoparticles (NPs), to combat both MDR bacteria and biofilm-forming *K. pneumoniae* is an acceptable and feasible alternative [7]. Accordingly, the use of green nanotechnology or green chemistry has the potential to improve the economy, health, and environment by supporting safer practices and reducing the production of potentially hazardous chemicals. In this context, the sustainable metabolites in plant extracts serve as bio-capping agents for synthesized nanoparticles [8].

In an effort to develop better antimicrobial agents for combating microbes that are resistant to numerous antimicrobial agents, the innovative use of antibacterial substances, including herbal products, has been increasing. Traditional treatments frequently involve medicinal products derived from aromatic herbs for managing bacterial infections [9]. Clove plants (*Syzygium aromaticum*) belong to the *Myrtaceae* family and are cultivated worldwide because of their aromatic and therapeutic properties [10]. Furthermore, it is widely recognized for its antibacterial, analgesic, anesthetic, anti-inflammatory, antioxidant, and insecticidal properties [11].

Therefore, the present study aimed to elucidate the prevalence of *K. pneumoniae* in cattle and humans, to determine the antibiogram and virulence-associated determinants and to evaluate the performance of incorporating Ag/Ni(OH)_2_/clove and Ag/Ni(OH)_2_/C nanocomposites synthesized using green technology to increase the effectiveness of these agents and minimize the potential side effects of the nanocomposites by assessing their cytotoxicity. Additionally, the antibiofilm activity of both Ag/Ni(OH)_2_/Clove and Ag/Ni(OH)_2_/C nanocomposites against biofilm-producing isolates of *K. pneumoniae* was investigated. In addition, computational studies are powerful tools that support scientific investigations at the atomic level [12,13]. Therefore, Monte Carlo (MC) simulations were carried out to evaluate the adsorption behavior of lipopolysaccharide (LPS), ergosterol, and chitin onto Ag/Ni(OH)_2_ composite as representative biomolecules of bacterial and fungal cell walls.

## 2. Materials and Methods

### 2.1. Study Location and Design

The current study was conducted on a private dairy farm in the Beni Suef region (coordinates 29°04′ N–31°05′ E), Egypt, from January 2025 to July 2025. A total of 130 samples were collected, including milk samples (*n* = 50) and feces (*n* = 50). Additionally, stool samples (*n* = 30) were taken from individuals admitted to the outpatient clinics at Beni Suef University (*n* = 27) and farm laborers (*n* = 3); all of them appeared apparently healthy during sample collection. Additionally, the University Higher Research Ethics Committee (BSU-HREC) at Beni Suef University certified the samples used in the study. *K. pneumoniae* was recovered by cultivating all of the samples and performing biochemical analysis followed by molecular confirmation. The disc diffusion technique was then used to evaluate the antibiotic sensitivity profile when seven drugs were used. Finally, a Congo Red (CRA) assay was used to investigate the development of biofilms. Finally, the effectiveness of the green synthesis of Ag/Ni(OH)_2_/Clove nanocomposites on isolates that exhibited multidrug resistance was evaluated using agar well diffusion, Minimal Inhibitory Concentration (MIC), and Minimal Bactericidal Concentrations (MBC) methods.

### 2.2. Sample Collection

A total of 130 samples were obtained under entirely aseptic conditions, comprising 50 milk, 50 feces and 30 stool samples. Oral permission from the farm manager was obtained before the animal samples were taken. Additionally, milk samples were taken by the milkers after pre-milking sanitation, and the first three streams of milk were thrown away. All the samples were collected in sterile plastic containers, and each sample was carefully labeled, sealed, and kept at 4 °C before being transported to the laboratory for bacterial culture and identification for less than twenty-four hours. Additionally, the Beni Suef University Higher Research Ethics Committee (BSU-HREC) approved the current study on 26 January 2025, with reference number HREC BSU\02612 at Beni Suef University approved for the human and animal samples included in the study. Additionally, informed consent for participation was obtained from all the subjects involved in the study.

### 2.3. Isolation and Identification of K. pneumoniae

Each sample was separately inserted into 10 mL of Tryptic Soy Broth (TSB), incubated for 18–24 h at 37 °C, cultured on the surface of MacConkey agar (MAC) plates, and incubated at 37 °C overnight. Pink mucoid dome-shaped colonies are indicative of lactose fermenters and are characteristic of suspected *Klebsiella* growth. The resulting *Klebsiella* isolates were subsequently purified on MacConkey agar and on Eosin Methylene Blue (EMB) agar plates. After that, a pure single colony was transferred to a tryptone soy agar (TSA) slant and maintained at 4 °C for biochemical examination utilizing Gram’s staining, motility, Simmons citrate agar, triple sugar iron, indole, and urease tests [14,15].

### 2.4. Molecular Identification of the Screened K. pneumoniae Isolates

A QIAamp DNA Mini kit (Qiagen GmbH, Germany) was used to extract DNA from each of the biochemically identified isolates. For amplification, a total volume of 25 µL, including 1 µL of 40 pmol of each primer, 12.5 µL of platinum Taq DNA polymerase, MgCl_2_ (Invitrogen), dNTPs, 6.5 µL of RNase-free water and 5 µL of target DNA, was used to amplify *K. pneumoniae*-specific genes. The following thermocycling parameters were used for the Applied Biosystems 2720 thermal cycler: a 5 min initial denaturation cycle at 94 °C, followed by 30 consecutive program cycles of 94 °C for 30 s, and an annealing temperature that varied between 53 and 63 °C for 45 s based on the amplified primer (Table 1). Additionally, 1.5% agarose (Applichem, Germany, GmbH) in 1× TBE buffer at room temperature with a gradient of 5 V/cm for 30 min was used to separate the secondary PCR products by gel electrophoresis. The gel was then observed and photographed with a UV transilluminator (Alpha Innotech, Biometra).

### 2.5. Antibiotic Sensitivity Profile

Seven types of antibiotics that are frequently used in clinical and animal treatments have been used to determine the susceptibility profiles of *K. pneumoniae* isolates (*n* = 56). The colonies were inoculated into TSB and incubated at 37 °C for 6 to 8 h. The broth turbidity used was 1.5 × 10^8^ colony-forming units per milliliter (CFU/mL), which is equal to 0.5 McFarland grade. After each sample was evenly spread over the Mueller Hinton agar (MHA, Sigma-Aldrich, Dorset, UK) surface, the antibiotic discs were placed, and the plates were incubated at 37 °C for 18–24 h. Following the instructions of the Clinical and Laboratory Standards Institute [19], the zone of inhibition encircling each disc was measured in millimeters (mm) and interpreted. The antibiotics employed in the study included amoxicillin/clavulanic acid (30 µg), sulfa-trimethoprim (SXT, 25 μg), fosfomycin (FOS, 20 μg), ciprofloxacin (CIP, 5 µg), amikacin (AK, 30 μg), gentamicin (GEN, 10 µg), and doxycillin (DO, 30 μg) (Oxoid, UK).

### 2.6. Biofilm Formation of K. pneumoniae Isolates

The Congo red (CRA) assay is a qualitative method for the detection of *K. pneumoniae* biofilm production [20]. The obtained bacterial strains (*n* = 56) were carefully inoculated onto Congo red agar plates and then incubated for 24–48 h at 37 °C. The recovered colonies were categorized as strongly positive (+++ve) when black colonies with a dry crystalline consistency appeared. Dark colonies with no dry crystalline morphology were interpreted as moderate positive biofilm producers (++ve), whereas colonies that did not change from pink were recorded as non-biofilm producers (−ve).

### 2.7. Preparation of the Extract

Fresh/dried clove buds (5 g) were used for the preparation of the clove extract. The cloves were washed thoroughly with distilled water, dried, and ground into a fine powder using a ball mill at 300 rpm for 15 min, with a ball-to-powder ratio of 10:1. The resulting material was mixed with 100 mL of distilled water and extracted at 50 °C for 60 min under continuous stirring. The extract was subsequently cooled and filtered using Whatman No. 1 filter paper to remove residual plant materials and insoluble particles [16]. The resulting filtrate was cooled to room temperature and used as the reducing agent. Until further use, the extract was stored at 4 °C and utilized within one week of preparation.

### 2.8. Synthesis of Ag/Ni(OH)_2_/Clove and Ag/Ni(OH)_2_/C Nanocomposites

The clove extract was used as both a reducing agent and a stabilizing agent for the synthesis of the nanocomposites. Briefly, equal volumes (25 mL each) of 1 M Ni(NO_3_)_2_ and 1 M AgNO_3_ solutions were added dropwise under continuous magnetic stirring to 50 mL of the prepared clove extract diluted in 100 mL distilled water, corresponding to a Ag:Ni molar ratio of 1:1. The reaction mixture was stirred continuously at room temperature for 2 h to allow complexation between the metal ions and the phytochemical constituents of the extract. The reaction mixture was adjusted to a pH of 6, which was sufficient to promote gradual hydrolysis and hydroxide formation. The mixture was then left undisturbed overnight for 6 h at room temperature under ambient conditions, after which the phytochemicals in the clove extract were allowed to reduce Ag^+^ ions while Ni^2+^ underwent controlled hydration and precipitation as Ni(OH)_2_. The resulting Ag/Ni(OH)_2_/clove nanocomposite was collected by centrifugation at 8000 rpm for 15 min, washed repeatedly with distilled water and ethanol to remove unreacted precursors and residual biomolecules, and dried in an oven at 60 °C for 6 h. The final product was obtained as a fine powder with a yield of approximately 85%.

The prepared material (Ag/Ni(OH)_2_/Clove nanocomposite) was transferred into a quartz boat placed inside a tube furnace. Thermal pyrolysis was conducted at 550 °C for 2 h under nitrogen at a heating rate of 5 °C/min. The pyrolyzed sample was subjected to a hydration process. The final product was obtained and collected as a Ag/Ni(OH)_2_/C nanocomposite.

### 2.9. Material Characterization

Crystallinity was analyzed using a PANalytical Empyrean X-ray diffractometer (Malvern Panalytical Instruments Ltd., Catalog Number; Empyrean, United Kingdom, Manufacturer: Malvern Panalytical B.V.; City: Almelo; Country: The Netherlands) with Cu-Kα radiation (λ = 0.154 nm) operated at 40 kV and 35 mA with a scanning speed of 1° min^−1^. The surface morphology and elemental composition were investigated using a Gemini Zeiss Sigma 500 VP field-emission scanning electron microscope (FE-SEM) coupled with energy-dispersive X-ray spectroscopy (EDS). Textural characteristics were evaluated through N_2_ adsorption–desorption measurements at 77 K using a Micrometrics TriStar II 3020 analyzer.

### 2.10. Antimicrobial Assessment of the Synthesized Ag/Ni(OH)_2_/Clove and Ag/Ni(OH)_2_/C Nanocomposites

#### 2.10.1. Preparation of the Bacterial Inoculum

The tested isolates were individually cultured in TSB (Oxoid, Catalog number: abx082157) for 18 to 24 h at 37 °C, and the turbidity was adjusted to 0.5 McFarland reaction or 1.5 × 10^8^ CFU/mL using phosphate-buffered saline (PBS, 0.9%).

#### 2.10.2. Preparation of Ag/Ni(OH)_2_/Clove and Ag/Ni(OH)_2_/C Suspensions

To achieve a final concentration of 1000 µg/mL, the synthesized bimetallic nanoparticles (0.01 g) were dissolved in 10.0% dimethyl sulfoxide (DMSO, Oxoid, Catalog Number: 102952; MerckTM, Germany) and then sonicated at a power of >20 kHz (Model: Q125 Sonicator^®^).

#### 2.10.3. Agar Well Diffusion Method

The prepared bacterial culture of 0.5 McFarland was evenly streaked over Mueller–Hinton agar (MHA; Sigma-Aldrich, Dorset, UK). After solidification, wells 6 mm in diameter were generated using a cork borer and 1000 µg/mL solutions of nanoparticles were distributed into the wells. Afterward, the plates were incubated for 24 h at 37 °C. The diameters were measured in mm, and the zone of inhibition (ZOI) was recorded. For every sample, three replicates were performed, and the mean was estimated [21]. Additionally, DMSO (10.0%) and tetracycline (30 µg/mL) were used as negative and positive controls, respectively.

#### 2.10.4. Determination of the Minimal Inhibitory Concentrations (MICs)

The biological effectiveness of the tested chemicals (1000 µg/mL) was evaluated against *K. pneumoniae* isolates using double-fold dilution methodology, followed by inoculation of each bacterial species in a volume of 100 µL of 1.5 × 10^8^ CFU/mL, after which the nanoparticle-bacterial broth mixture was incubated for 24 h at 37 °C. The lowest antimicrobial agent concentration that absolutely suppresses noticeable growth of bacteria in the tubes is known as the minimum inhibitory concentration (MIC). Additionally, aliquots of approximately 10 µL of solution from each tube that did not exhibit turbidity were seeded on TSA plates and incubated for 24 h at 37 °C. The minimum bactericidal concentration (MBC) threshold is defined as the concentration at which 100.0% of the bacterial population is eliminated at the lowest concentration of an antimicrobial agent [22]. All the MIC and MBC experiments were repeated in triplicate, and the mean was determined. Additionally, the bactericidal or bacteriostatic evidence of the tested compounds was determined by the MBC/MIC ratios according to the following equation:

$$Tolerance/Bactericidal\;Ratio=\frac{Minimum\;Bactericidal\;Concentrations\;of\;the\;tested\;chemical\;(MBCs)}{Minimum\;Inhibitory\;Concentrations\;of\;the\;tested\;chamical\;(MICs)}$$

A ratio between 1 and 2 indicated a bactericidal effect, where values higher than 4 indicated static potency [19].

#### 2.10.5. Antibiofilm Potency of the Tested Chemicals

Ag/Ni(OH)_2_/Clove and Ag/Ni(OH)_2_/C nanocomposites were tested for their antibiofilm properties against 15 *K. pneumoniae* isolates that had previously demonstrated biofilm formation in CR agar media. In short, the isolates were cultivated on Tryptone soya agar for 18 h at 37 °C, and the colonies were inoculated in saline (NaCl, 0.9%) to create a 0.5 McFarland equivalent. CRA media was then prepared, sterilized in an autoclave for 15 min at 121 °C, and left to cool at 55 °C. The tested nanomaterials at concentrations of 100, 500, 750, and 1000 μg/mL were mixed with CRA media. After being placed into sterile Petri dishes, the nanomaterial-agar medium was left to solidify. The bacterial suspensions were streaked onto the media surface, and the plates were incubated at 37 °C for 24–48 h to evaluate the color of the colony growth [20]. Additionally, a negative control was employed, which consisted of strains that were cultured on CRA medium that was devoid of nanomaterials.

#### 2.10.6. Assessment of Cytotoxicity to HEK293 Cells

The cytotoxic effects of synthesized Ag/Ni(OH)_2_/Clove and Ag/Ni(OH)_2_/C nanocomposites were evaluated in vitro using human embryonic kidney (HEK293) cells through the MTT assay. The HEK293 cell line was obtained from the ATCC (Manassas, VA, USA; catalog number. CRL-1573). This assay determines cell viability by measuring the formation of formazan, a colored compound produced when metabolically active cells reduce MTT [23], in his seminal work on the MTT assay methodology. HEK293 cells were maintained in Dulbecco’s modified Eagle’s medium (DMEM) enriched with 10.0% fetal calf serum and 1.0% antibiotic-antimycotic solution and incubated at 37 °C with 5.0% CO_2_ in a humidified environment, following standard cell culture protocols [24]. For the experiments, the cells were plated in 96-well plates at a density of 1 × 10^5^ cells per well and allowed to adhere for 24 h. Afterward, the medium was replaced with fresh DMEM containing various concentrations (7.8–1000 μg/mL) of the prepared nanoparticles, with treatments performed in triplicate, and the cells were incubated for another 24 h. Following exposure, the cells were washed three times with phosphate-buffered saline (PBS), and 20 μL of MTT solution (5 mg/mL) was added to each well, allowing for a 4 h reaction period, as optimized by Berridge et al. [25] for accurate cytotoxicity assessment. After incubation, the medium and residual MTT were removed, and 100 μL of dimethyl sulfoxide (DMSO) was added to dissolve the formazan crystals. The plate was gently mixed for 15 min to ensure full dissolution, and the absorbance was measured at 570 nm using an MRX microplate spectrophotometer, which is consistent with the protocol outlined by Stockert et al. [26] for formazan quantification.

### 2.11. MC Simulation

In this study, the BIOVIA Materials Studio 2024 package was used to investigate the adsorption mechanism of LPS, ergosterol, and chitin [27] molecules on the Ag/Ni(OH)_2_ surface via MC simulation. Ag/Ni(OH)_2_ of the (010) surface has parameters a = 37.2 Å, b = 37.888 Å, and c = 47.812 Å, and the lattice angles α = 90°, β = 90°, and γ = 90°. Moreover, Ag/Ni(OH)_2_ of the (100) surface has parameters a = 34.375 Å, b = 37.5 Å, and c = 52.659 Å, and the lattice angles α = 90°, β = 90°, and γ = 120°. The system has a sufficient spatial spacing of 40 Å along the direction perpendicular to the surface. Using the Forcite module in Materials Studio, the geometry of the molecules and surfaces was optimized before the MC simulations were performed at 300 °K. Universal force fields, current charges, Ewaild electrostatic interactions, and atom-based ven der Waals interactions were used in the geometry optimization and MC simulations. For each system, 3 cycles were performed, including 45,000 optimization steps.

### 2.12. Statistical Analysis

Statistical software for social sciences (SPSS, Inc., version 22.0; Chicago, IL, USA) was used to evaluate the frequency of *K. pneumoniae* isolated from the studied samples and the antibiotic sensitivity profile using nonparametric tests (chi-square test). Additionally, a t test was used to determine the antibacterial efficacy of the synthesized nanomaterials, and the results are presented as the means ± SEs, where * indicates a significant difference at *P* ˂ 0.05.

## 3. Results and Discussion

The steps involved in preparing the nanocomposites are shown in Figure 1. During the mixing process, the biomolecules present in the clove extract effectively reduced Ag^+^ ions to metallic silver nanoparticles (Ag NPs) while simultaneously coordinating with Ni^2+^ ions to form a stable bioorganic capped precursor framework. During carbonization, the organic matrix was thermally converted into a conductive carbon support, while the metal species concurrently reduced the metal components to form Ag/Ni-based nanocomposite. Following thermal treatment, the pyrolyzed sample was subjected to a hydration process. Owing to the high surface energy and hygroscopic nature of the newly formed nanoscale components, a surface hydration reaction took place, which transformed the surface-exposed nickel species into stable Ni(OH)_2_.

Comparative XRD patterns of the synthesized Ag/Ni(OH)_2_/Clove and Ag/Ni(OH)_2_/C nanocomposites demonstrating characteristic reflections are presented in Figure 2a. In Ag/Ni(OH)_2_/Clove nanocomposite, the XRD analysis revealed that the clove extract is an amorphous organic material, characterized by a broad intensity hump indicating the presence of organic compounds. Moreover, several weak peaks were observed in the sample because of the accumulation of organic matter. However, after carbonization of Ag/Ni(OH)_2_/C nanocomposite, the crystallinity increases, as evidenced by the height of the peaks and the prominence of the carbon peaks. Furthermore, crystalline phases of Ag particles were identified through distinct peaks at 37.62°, 43.66°, 63.22°, and 76.74°, corresponding to the (111), (200), (220), and (311) planes, respectively [28,29]. Concurrently, Ni(OH)_2_ phase was confirmed by diffraction peaks at 2θ values of 19.82°, 32.3°, 32.3°, 38.46°, 52.18°, 62.7°, and 74.18°, corresponding to the (111), (100), (002), (012), (110), (220), and (201) Miller indices, respectively [30,31].

Additionally, the average crystallite size decreased slightly for Ag/Ni(OH)_2_/C nanocomposite, from 40.80 nm for the as-prepared sample to 39.2 nm after carbonization of Ag/Ni(OH)_2_/C (Figure 2b), indicating that the crystallite size decreased as the carbonization temperature increased in the presence of the carbon matrix. This marginal decrease in crystallite size can be attributed to thermal degradation and subsequent removal of the organic matrix (clove extract) intercalated within the layers. The expulsion of these organic species induces strucxzedcftural contraction and lattice reconfiguration, hindering further grain growth and leading to slight grain refinementduring the carbonization process [32]. Moreover, XRD analysis revealed important changes in the average micro-strain distribution among the samples, with calculated values increasing from 0.34082% for the as-prepared sample to 0.54782% for Ag/Ni(OH)_2_/C, as shown in Figure 2c. This pronounced increase in micro-strain in Ag/Ni(OH)_2_/C nanocomposite can be attributed to the thermal stress induced during the heating process, along with the promotion of crystallite growth and potential lattice distortions. As the carbonization process drives the expulsion of volatile organic components and triggers phase transformations, it introduces microscopic dislocations and defects within the crystal lattice, thereby increasing the internal micro-strain. Furthermore, this structural reorganization often leads to a contraction or expansion of the unit cell volume directly impacting the peak broadening observed in the XRD patterns [32,33].

As shown in Figure 2d, the FTIR spectrum of Ag/Ni(OH)_2_/Clove nanocomposite (before carbonization) displayed broad and intense absorption bands at 3394 and 3203 cm^−1^, which are characteristically assigned to the stretching vibrations of the hydroxyl (-OH) groups and intercalated water molecules [34,35]. The presence of clove extract (primarily eugenol) within the matrix is confirmed by distinct organic functional bands. Specifically, the sharp absorption peaks at approximately 2927 and 2854 cm^−1^ correspond to the asymmetric and symmetric stretching vibrations of aliphatic C-H groups, respectively [36,37]. Furthermore, the prominent band observed at 1627 cm^−1^ is attributed to the bending vibration of intercalated water molecules, potentially overlapping with the aromatic C=C stretching modes of the phenolic rings in the clove extract [36,37]. The absorption peaks in the fingerprint region, which span 1514 and 1400 cm^−1^, are indicative of aromatic ring vibrations and C-H deformation, respectively. Additionally, the characteristic C-O-C ether linkage of eugenol appears at approximately 1269 cm^−1^ [38]. These results confirm the successful preparation of the clove-based nanocomposite. At lower wavenumbers, the peaks at 918, 848, and 817 cm^−1^ are assigned to the lattice vibrations of Ag bonds [39,40]. Moreover, the peaks at 644 and 435 cm^−1^ are assigned to the lattice vibrations of Ni bonds [41,42]. These results confirm the successful coordination between Ag/Ni(OH)_2_ and the organic matrix.

In the FTIR spectrum of Ag/Ni(OH)_2_/C nanocomposite, the aliphatic C–H expansion bands (2927 and 2854 cm^−1^) and the specific ether bands (1269 cm^−1^) of the eugenol matrix disappeared as a result of thermal decomposition. However, instead of the complete disappearance of carbonaceous features, distinct features emerged. A broad, weak band appeared at 1618 cm^−1^, which is attributed to C=C stretching vibrations of the aromatic carbon sheets, confirming the carbonization of the clove extract into an amorphous carbonaceous structure. Moreover, the sharp decrease in the intensity of the -OH band (3419 and 3143 cm^−1^) confirms the presence of hydroxylated layers. In the low-wavenumber region, the peaks of Ag bonds shifted to 871 and 833 cm^−1^, whereas the peaks of Ni bonds shifted to 671 and 468 cm^−1^. New intense bands associated with Ag/Ni(OH)_2_/Clove nanocomposite lattice vibrations appeared, indicating the formation of metal hydroxide anchored within the formed carbon matrix. This carbonization behavior is consistent with the XRD findings. The pyrolysis of clove extract in an inert atmosphere leads to the formation of an amorphous carbon matrix (as evidenced by the broad XRD pattern and the C=C FTIR band). This in situ-formed carbon skeleton acts as a physical barrier that confines Ni/Ag species, restricting their agglomeration and resulting in the observed grain refinement (39.20 nm). Consequently, the encapsulation of Ag/Ni(OH)_2_/C nanocomposite phases within this rigid carbon network induces significant lattice mismatch and thermal mismatch stress, directly accounting for the pronounced increase in the micro-strain to 0.54782%.

Scanning electron microscopy (SEM) was used to generate three-dimensional images of the sample surfaces, revealing microscopic and nanoscale details and structural details of Ag/Ni(OH)_2_/Clove nanocomposite and Ag/Ni(OH)_2_/C nanocomposites, as shown in Figure 3. In Figure 3a, micrometer-scale spacing and the formation of large pores with deep wrinkles within the clusters can be observed. In Figure 3b,c, small wrinkles in nanoscale dimensions and the surface roughness characteristics of the clove-derived material can be observed. Additionally, nanoparticles of varying sizes are distributed across the surfaces, which is characteristic of Ag/Ni(OH)_2_/Clove nanocomposite. As shown in Figure 3d, the homogeneity observed throughout Ag/Ni(OH)_2_/C nanocomposite sample, with a textured surface at the microscale, indicates that heat treatment was distributed and increased the surface area. In Figure 3e,f, numerous nanopores were observed within the depth of the surfaces. Moreover, nanosheet-like structures on the surfaces were observed and attributed to Ni(OH)_2_. Thus, heat treatment improved the nanoscale morphology and surface area, which enhanced the efficiency of the materials.

To further investigate and highlight these differences, the 3D surface topography and surface roughness were examined, as shown in Figure 4. Ag/Ni(OH)_2_/Clove nanocomposite has heterogeneous surfaces and agglomerations (Figure 4a,b), whereas Ag/Ni(OH)_2_/C nanocomposite is homogeneous across its surface (Figure 4c,d). The surface roughness indicates the internal porosity throughout the material as well as the textured and shattered surfaces. Moreover, the porous nanomaterials exhibit a highly ordered surface structure, Ag/Ni(OH)_2_/C, characterized by a complex, interconnected network of nanopores. This surface structure creates a large surface-to-volume ratio with well-defined pore geometries that govern permeability and interfacial interactions.

Energy-dispersive X-ray spectroscopy (EDX) was employed to determine the elemental and chemical compositions of Ag/Ni(OH)_2_/Clove and Ag/Ni(OH)_2_/C nanocomposites, as shown in Figure 5a,b. The EDX spectra exhibited characteristic peaks associated with Ni, Ag, Cl, C, and O. The weight percentages of C, O, and Cl decreased in the Ag/Ni(OH)_2_/C sample from 31.88%, 6.88%, and 18.67% to 22.93%, 3.16%, and 9.25%, respectively. Moreover, Ni and Ag increased in Ag/Ni(OH)_2_/C sample from 23.96% and 18.61% to 36.28% and 28.38%, respectively.

Zeta potential analysis revealed significant differences in physical stability and surface charge between Ag/Ni(OH)_2_/Clove and Ag/Ni(OH)_2_/C nanocomposites. This behavior is related to the difference in electrical conductivity of the solution. As shown in Figure 5c, Ag/Ni(OH)_2_/Clove nanocomposite exhibited a conductivity of 1.89 mS/cm, which caused electrostatic shielding and compression of the bilayer, reducing the zeta potential to −8.55 mV with a standard deviation of 4.82 mV. This resulted in weak electrostatic repulsion forces and a tendency for the suspension to aggregate and settle rapidly. In contrast, the low conductivity of 0.797 mS/cm in Ag/Ni(OH)_2_/C nanocomposite sample contributed to reducing the number of free ions (Figure 5d), which allowed the bilayer to expand, producing a strong positive surface charge of +38.3 mV with a standard deviation of 5.78 mV, which exceeds the critical limit for colloidal stability (>30 mV) and confirms the excellent stability of the suspension [43]. Here, the zeta potential distribution shows that heat increased the surface charge, which increased the porosity, stability, and surface area and reduced the degree of agglomeration.

According to our results, *K. pneumoniae* was recovered from the stool, milk, and fecal samples at rates of 43.3%, 38.0%, and 48.0%, respectively (Table 2). Lower isolation rates of *K. pneumoniae* were reported by [44] 41% (16/39), [45] (35.48%) and [46] (26.94%) in fecal samples. Additionally, ref. [47] (6.5%), ref. [48] (10.52%), and ref. [49] (25.7%) identified *K. pneumoniae* from milk samples. Furthermore, ref. [50] reported that 35 (39.0%) of the tested patients had *K. pneumoniae* isolated, whereas refs. [51,52] reported similar isolation rates of *K. pneumonia* in 30.0% of stool samples.

In the current study, the higher recovery rates of *K. pneumoniae* in the fecal samples may have been previously linked to the abundance of organic matter in the farm environment. Moreover, deficient cleaning procedures were performed on the animal surroundings or on the teats before and after milking. Furthermore, it is known that cattle are reservoirs for *K. pneumoniae* [53]. This is linked to the inconsistent fecal shedding of *Klebsiella*, which has been observed in up to 80.0% of healthy dairy cows [54,55]. Furthermore, *K. pneumoniae* represents one of the predominant causes of acutely acquired environment-related infections [56]. This poses a serious threat to public health, particularly for farm workers, as it can disseminate via the fecal–oral route, leading to substantial morbidity and potentially fatal infections such as pneumonia, bloodstream and urinary tract infections, as well as sepsis [57], especially in individuals with compromised immune systems [57].

Antimicrobial susceptibility testing revealed no significant pattern of resistance across the different antibiotic categories (Table 3). The majority of *K. pneumoniae* isolates exhibited a similar pattern of resistance to certain antibiotics: isolates from fecal samples shared resistance to sulfa trimethoprim, doxycycline, amoxicillin/clavulanic acid, and ciprofloxacin (75.0% each), as did isolates of *K. pneumoniae* from milk samples against sulfa trimethoprim, gentamicin, and doxycycline (73.7% each), as did those from stool samples against sulfa-trimethoprim, amikacin, and gentamicin (76.9% each). Additionally, moderate resistance was observed for *K. pneumoniae* isolated from fecal samples against amikacin (50.0%) and gentamicin (58.3%) and for milk and stool isolates against ciprofloxacin (57.9% and 53.8%, respectively). Notably, our findings revealed that most *K. pneumoniae* isolates from stool, milk, and fecal samples exhibited multidrug resistance (MDR), defined as resistance to two or more drug categories.

Numerous studies have revealed that *K. pneumoniae* is isolated from strains with variable levels of resistance to various classes of antibiotics. For example, ref. [58] reported that *K. pneumoniae* in mastitic milk was sensitive to ciprofloxacin and gentamicin. According to [47], *K. pneumoniae* isolates are sensitive to sulfa-methoxazole/trimethoprim, ciprofloxacin, amikacin, gentamicin, tetracycline, and oxytetracycline. Additionally, ref. [52] reported that *K. pneumoniae* isolates were resistant to ceftazidime (80.0%), nalidixic acid (33.0%), amikacin (36.0%), azithromycin (20.0%), vancomycin (10.0%), and imipenem (6.0%) when they were recovered from stool samples. According to [59], *K. pneumoniae* isolates recovered from nasal swab samples of cattle in China that exhibited respiratory symptoms presented relatively high resistance to ciprofloxacin but relatively low resistance to ceftazidime and amikacin. Additionally, ref. [44] reported that all strains of *K. pneumoniae* from calves suffering from diarrhea had 100.0% resistance to erythromycin, azithromycin, and amoxicillin, indicating MDR. Furthermore, ref. [45] reported that *K. pneumoniae* cultivated from bovine diarrhea in India had a very high percentage of resistance to tetracycline (75.0%), followed by that to gentamicin, streptomycin, and ceftriaxone (50.0% each), while a low percentage of the isolates were resistant to enrofloxacin (37.50%), and the lowest proportion were resistant to amikacin (25.0%).

Increased *Klebsiella* carriage in the intestinal tract of humans and animals and the emergence of multidrug-resistant strains resulted from the extensive use of many classes of antibiotics in clinical cases of gastrointestinal infections, mastitis, and other illnesses in the study area. Additionally, the capsule provides strong protection *for K. pneumoniae*, a multidrug resistance efflux pump that is effective at acquiring and spreading resistance plasmids, and the spread of antibiotic resistance among humans, animals, and environments, all of which have important implications for the one-health approach [59,60].

A total of 56 *K. pneumoniae* isolates from animal and human samples were tested using the CRA method to assess biofilm production (Table 4). The results revealed that 22 isolates strongly formed biofilms (39.3%), 15 isolates moderately formed biofilms (26.8%) and 8 isolates weakly formed biofilms (14.3%). However, 11 of the collected *K. pneumoniae* isolates (19.6%) failed to develop biofilm characteristics. In contrast, ref. [61] reported that 93.6% of *K. pneumoniae* strains in Iran produced biofilms; in addition, in an earlier study conducted in Nepal, ref. [62] reported that 73.3% of *K. pneumoniae* strains produced biofilms. Moreover, all the strains of *K. pneumoniae* were shown to be biofilm formers [63]. Furthermore, according to [64], 91.7% of *K. pneumoniae* isolates were able to form biofilms, whereas 8.3% were unable to do so. Six (54.5%), three (27.3%), and two (18.2%) of the 11 *K. pneumoniae* isolates produced biofilms in moderate, strong, and weak manners, respectively. Our observations demonstrated a clear correlation between biofilm production and antibiotic resistance in *K. pneumoniae* strains, which enables the bacterial cells to encase themselves in an extracellular matrix of proteins, DNA, and polysaccharides. The presence of As highlighted by the presence of both aerobactin (*iut*-A gene, amplifying 300 bp fragment) and type III fimbriae (*mrk*-A gene, amplifying a 475 bp fragment) in the tested isolates of animal and human origin (Figure 6) increases resistance to antimicrobial substances and stress conditions, plays a crucial role in pathogenicity, and is an integral virulence mechanism [65].

Globally, treatment failure and mortality rates have increased because of the increasing resistance of *K. pneumoniae*, especially those that produce biofilm characteristics [66]. Consequently, environmentally friendly, nonhazardous, sustainable compounds with strong therapeutic applications are produced by the green synthesis of metal nanoparticles [67]. Additionally, our findings revealed that *K. pneumoniae* strains exhibited variable sensitivities to Ag/Ni(OH)_2_/Clove nanocomposite-calcined form to differing degrees, according to the inhibition zone, MIC, and MBC at a concentration of 1000 μg/mL. The calcined formula demonstrated strong and superior bactericidal efficacy, as evidenced by greater zones of inhibition (20.67 ± 1.76 mm) and lower MIC and MBC values (20.83 ± 5.23 μg/mL) (36.50 ± 13.81 μg/mL), with MBC/MIC values lower than 4 (Table 5). Furthermore, the efficacy of Ag/Ni(OH)_2_/Clove and Ag/Ni(OH)_2_/C nanocomposites in preventing biofilm development against strains of *K. pneumoniae* cultured on CRA at 37 °C for 24 h with varying concentrations of the nanoparticles (100, 500, 750, and 1000 μg/mL) was evaluated (Table 5). There was an apparent dose-dependent inhibitory influence of both materials on biofilm formation, where the formation of biofilms decreased gradually as the concentration of the nanomaterials increased, where the most potent antibiofilm effect was observed at a concentration of 1000 μg/mL for Ag/Ni(OH)_2_/C nanocomposites (86.7%) in comparison to Ag/Ni(OH)_2_/Clove nanocomposites (73.3%).

Data reported by Bhat et al. [68] revealed that silver nanoparticles combined with *Citrus limon* demonstrated antimicrobial efficacy against *K. pneumoniae* isolates, with an inhibition zone of 14 ± 1.5 mm, an MIC of 15.6 μg/mL, and an MBC of 62.5 μg/mL. Similarly, the conjugation of silver nanoparticles with *Citrus sinensis* resulted in comparable observations, with an inhibition zone of 16 ± 1.0 mm, an MIC of 7.81 μg/mL, and an MBC of 31.25 μg/mL. Additionally, the antibacterial activity of AgNPs against *K. pneumoniae* was reported by [69], with MIC values ranging from 15.625 μg/mL to 125 μg/mL. Similarly, the bactericidal effectiveness of clove essential oils was demonstrated against *K. pneumoniae* isolates, with inhibition zones ranging from 15.0 ± 1.00 to 19.0 ± 1.00 mm, an MIC value of 7.81 μg/mL, an MBC standard of 15.6 μg/mL, and an MBC/MIC ratio of two. Additionally, ref. [70] reported that the synthesis of NiO NPs utilizing an ecologically safe method in the leaf extract of *Populus ciliata* resulted in strong antibacterial activity against strains of *K. pneumoniae,* with an inhibition zone of 12.2 ± 0.3 mm. Furthermore, at a concentration of 100 μg/mL, Ni nanoparticles prepared with *Ocimum sanctum* leaf extract showed outstanding biological activity against *K. pneumoniae* [71].

The presence of Ag ions in the synthesized formulation is the main cause of the toxic effects of Ag NPs, as these ions disrupt the respiratory chain of *K. pneumoniae* and attach to sulfhydryl bonds in the membrane of bacterial cells and vital enzymes [72]. Additionally, reactive elements such as radicals or oxidants, which are frequently found on the exterior structures of nanoparticles, promptly participate in the formation of ROS, which causes oxidative stress and the death of cells [73], with minimal cytotoxicity and no triggering of an immune response [74]. Additionally, the antibacterial properties of Ni NPs are related to the presence of Ni ions, which contribute to the electrostatic attraction between positively charged Ni particles and negatively charged microbial cells. This allows Ni to enter the bacterial cell membrane and reach intracellular organelles, where it damages ribosomes and affects bacterial metabolism. Additionally, Ni ions strongly attract phosphorus and sulfur in bacterial pathogens, such as proteins and DNA, thereby disrupting DNA replication and impairing protein synthesis [75]. In addition, clove plants contain a number of bioactive chemicals, including eugenol (70.0–85.0%), which represents 70.0–85.0%, eugenyl acetate (15.0%), and β-caryophyllene (5.0–12.0%), which are related to bactericidal effects on bacteria, inducing cell membrane defects and pore formation in their protective envelope, leading to bacterial death [76].

The phenotypic characteristics of the bacterial colonies underwent a transformation from black to pink or red, which was indicative of the complete inhibition of bacterial clustering and the assembly of the matrix-associated exopolysaccharides (EPS), which are crucial steps in biofilm biomass production [77]. However, the negative controls inoculated on nanoparticle-free media developed black, dry, crystalline colonies. These findings suggest that the clustering of EPS is essential for biofilm production. The high concentration of phenolic compounds in clove, such as eugenol, is frequently linked to its antimicrobial and antibiofilm activities against a number of pathogen-related infections, such as those caused by *Escherichia coli* O157:H7, *Listeria monocytogenes*, *Staphylococcus aureus*, *Candida albicans*, *Pseudomonas aeruginosa*, and *K. pneumoniae*. This is because eugenol can destabilize the biofilm matrix and interfere with the quorum sensing system, which controls bacterial communication within the biofilm biomass [78,79]. Furthermore, AgNPs exhibit potent antibacterial and antibiofilm properties by lysing cell membranes, generating reactive oxygen species (ROS), and disrupting ribosomes, mitochondria, DNA, and RNA, as well as interfering with cellular pathways [80]. Therefore, the combination of silver and nickel metallic nanoparticles in the clove nanoformulation demonstrated multifunctional properties that enhanced their effect against biofilm-producing strains of *K. pneumoniae*.

The cytotoxic effects of Ag/Ni(OH)_2_/Clove and Ag/Ni(OH)_2_/C nanocomposites on HEK293 cells were assessed using the MTT assay. As presented in Figure 7, the results demonstrated a clear concentration-dependent reduction in cell viability following 24 h of exposure, which is consistent with findings from previous studies on metal nanoparticle cytotoxicity [81]. At lower concentrations (7.8–62.5 µg/mL), both nanoparticle formulations exhibited minimal cytotoxicity, maintaining cell viability above 90.0%, thereby indicating good biocompatibility within this range, similar to observations reported for green-synthesized nanoparticles [82,83]. Across the 7.8–500 µg/mL range, the cell viability of the calcined nanoparticles (Ag/Ni(OH)_2_/C) was consistently slightly greater than that of the non-calcined nanoparticles (Ag/Ni(OH)_2_/Clove); for example, at 125 µg/mL, the viability was 84.62 ± 2.1% for Ag/Ni(OH)2/C versus 79.18 ± 1.3% for Ag/Ni(OH)_2_/Clove (*P* = 0.026, Welch’s t test; Table 6), which is consistent with reports that calcination reduces surface reactivity and aggregation, thereby mitigating cytotoxicity [84]. At the highest concentration tested (1000 µg/mL), both formulations markedly reduced viability, and the trend reversed: the non-calcined sample retained slightly higher viability (16.38 ± 2.55%) than the calcined sample did (12.09 ± 1.32%), although this difference was not statistically significant (*P* = 0.081). The calculated IC_50_ values were 264.7 µg/mL for Ag/Ni(OH)_2_/Clove and 282.8 µg/mL for Ag/Ni(OH)_2_/C, indicating a marginally higher tolerated dose for the calcined formulation overall, which is consistent with the low-to-mid concentration trend. These findings suggest that both nanoparticle formulations exhibit acceptable biocompatibility at lower concentrations, supporting their potential applicability in biomedical fields, as highlighted by [85] in their review on biogenic nanoparticles [86]. Furthermore, the enhanced cytocompatibility of the calcined nanoparticles at low-to-moderate concentrations highlights the beneficial impact of calcination on reducing cytotoxicity and improving safety profiles for future biological applications, as corroborated by research on the effects of thermal treatment on nanoparticle biocompatibility; the convergence of the two formulations at the highest tested dose indicates that this benefit is concentration-dependent and should not be extrapolated beyond the tested range without further mechanistic study.

The adsorption behavior of lipopolysaccharide (LPS), ergosterol, and chitin on Ag/Ni(OH)_2_ surfaces with (010) and (100) crystallographic orientations was investigated using molecular simulation. These biomolecules were selected because of their essential roles in microbial structure and function, where LPS represents a key component of the outer membrane of Gram-negative bacteria [87], ergosterol is a fundamental sterol in fungal cell membranes [88,89], and chitin constitutes a major structural component of the fungal cell wall, as shown in Figure 8 [90,91].

The equilibrium configurations obtained from the Monte Carlo simulations are shown in Figure 9. The corresponding average total energy, adsorption-energy profiles, and energy distributions are presented in Figure 10, Figure 11, Figure 12 and Figure 13, respectively, while the spatial characteristics of the adsorbate–surface interactions are further examined using RDF analysis, as shown in Figure 13. The optimized structures suggest that adsorption occurs through multiple interaction sites depending on the functional groups of each molecule, as shown in Figure 9. Ergosterol interacts via its rigid hydrophobic steroid backbone. Chitin [92]. LPS is expected to interact primarily through its phosphate and hydroxyl groups [93]. On the other hand, interacts mainly through hydroxyl and acetamido functional groups. These diverse interaction modes indicate that Ag/Ni(OH)_2_ surfaces are capable of forming strong and multisite interactions with biologically relevant molecules.

The average total energy profiles obtained during the Monte Carlo simulations are shown in Figure 10. The average total energy of LPS, ergosterol, and chitin initially decreases rapidly during the early simulation steps and approaches a relatively stable region with small fluctuations. This behavior indicates that the adsorption systems evolved toward energetically stable configurations during the Monte Carlo sampling. The initial decrease in total energy reflects the relaxation of the adsorbate–surface configurations toward more favorable arrangements. The subsequent fluctuations around a relatively stable energy region indicate the attainment of an equilibrated state. The average total energy of LPS, ergosterol, and chitin decreases to 231.12, 67.18, and 3155.08 kcal/mol, respectively, on the (010) surface. Moreover, it decreases to 226.7, 65.5, and 3154.66 kcal/mol on the (100) surface, respectively. The similar stabilization behavior observed for both the (010) and (100) surfaces supports the reliability of the sampled equilibrium configurations used for the subsequent adsorption-energy, energy-distribution, and RDF analyses.

Adsorption calculations revealed six distinct equilibrium configurations corresponding to the interaction of each biomolecule with the two crystallographic surfaces, as shown in Figure 9. Changes in the configurations were recorded during the optimization processes at several phases. The results indicate that the adsorption behavior is strongly dependent on the surface orientation, suggesting that variations in the atomic arrangement and electronic environment of the exposed facets significantly influence the interaction strength and adsorption geometry (Figure 10). For LPS, the calculated adsorption energies ranged from −45.80 to −50.15 kcal/mol, whereas the corresponding rigid adsorption energies ranged from −30.90 to −32.23 kcal/mol for the (010) and (100) surfaces, respectively. The highly favorable adsorption of LPS can be attributed to its complex molecular structure, which contains multiple phosphate, hydroxyl, and oxygen-containing functional groups that provide numerous sites for interactions with the metal centers and hydroxyl groups on Ag/Ni(OH)_2_ surface. The more negative adsorption energy obtained for the (100) surface indicates a more favorable interaction with LPS on this facet, which may be associated with the greater accessibility and spatial distribution of surface-active sites. In contrast, the adsorption energies of ergosterol were moderate, ranging from −21.44 to −23.46 kcal/mol, with corresponding rigid adsorption energies of −21.58 to −23.80 kcal/mol on the (010) and (100) surfaces, respectively. Although these interactions are weaker than those calculated for LPS, the negative adsorption energies indicate favorable interactions between Ag/Ni(OH)_2_ surface and ergosterol. From a computational perspective, these results suggest a potential interaction of Ag/Ni(OH)_2_ surface with ergosterol, which may be relevant to its predicted interaction with fungal membrane components. However, the results of the adsorption calculations alone do not provide experimental evidence of antifungal activity. Chitin exhibited the weakest adsorption among the three biomolecules, with adsorption energies of −8.82 and −8.08 kcal/mol and rigid adsorption energies of −8.84 and −8.18 kcal/mol on the (010) and (100) surfaces, respectively. The relatively weak interactions may be associated with the limited accessibility of reactive functional groups within the chitin structure and the reduced possibility of forming strong electrostatic or coordination interactions with the surface. Notably, the (010) surface exhibited slightly more favorable adsorption toward chitin than the (100) surface did, indicating that the effect of crystallographic orientation is biomolecule-dependent rather than universally favorable for one particular facet.

Thus, on the basis of the magnitude of the calculated adsorption energies, the adsorption strength follows the order of LPS > ergosterol > chitin for both crystallographic orientations. The substantially more negative adsorption energies obtained for LPS indicate a strong affinity of Ag/Ni(OH)_2_ surface toward this bacterial outer-membrane component, which is particularly relevant to Gram-negative bacterial systems. In contrast, the weaker but still favorable interactions calculated for ergosterol and chitin should be regarded as computational predictions of potential interactions with fungal membrane and cell-wall components, respectively, rather than direct evidence of antifungal activity. The adsorption results therefore support the proposed antimicrobial interaction of Ag/Ni(OH)_2_ with bacterial membrane components, while the potential interactions with fungal targets require experimental antifungal validation. The differences in adsorption behavior between the two crystallographic orientations further demonstrate that surface structure plays an important role in determining biomolecule–surface interactions. With respect to LPS and ergosterol, the (100) surface had more favorable adsorption energies, whereas the adsorption of chitin was slightly more favorable on the (010) surface. These results indicate that the adsorption behavior is governed by the specific molecular structure and its spatial arrangement relative to the surface-active sites. Moreover, adsorption energy and radial distribution function (RDF) analyses provide complementary information: the adsorption energy reflects the overall energetic favorability of the adsorbed state, whereas the RDF describes the spatial distribution and preferred separation distances between the interacting species. Consequently, differences between the adsorption-energy and RDF trends do not necessarily indicate contradictory behavior but rather reflect different aspects of the adsorption process (Figure 11a–d).

Energy distribution analysis further characterized the adsorption behavior of the investigated biomolecules on Ag/Ni(OH)_2_ surfaces, as shown in Figure 12. In Figure 12a, the adsorption systems involving LPS exhibited the most negative energy distributions, extending from −51.55 to −15.85 kcal/mol for the (010) surface and from −56.25 to −16.05 kcal/mol for the (100) surface. The substantial population of configurations within the negative-energy region indicates favorable adsorption states and stabilization of LPS on both surfaces. The broader distribution and its extension toward more negative energies for the (100) surface are consistent with its more favorable calculated adsorption energy, suggesting a more favorable energetic environment for LPS–surface interactions. For ergosterol adsorption (Figure 12b), the energy distributions extended from −25.35 to 0.15 kcal/mol for the (010) surface and from −25.95 to 0.15 kcal/mol for the (100) surface. The less negative energy range compared with that of LPS indicates a lower degree of energetic stabilization. This behavior can be associated with the predominantly hydrophobic steroidal structure of ergosterol and its smaller number of strongly polar functional groups, which may limit the formation of strong electrostatic or coordination interactions with the active sites of Ag/Ni(OH)_2_ surface. These results suggest a favorable interaction between ergosterol and Ag/Ni(OH)_2_ surface. However, they should be interpreted as a prediction of potential interactions with a fungal membrane component rather than direct evidence of antifungal activity. In contrast, the chitin adsorption systems exhibited the narrowest and least negative energy distributions, ranging from −11.95 to 0.15 kcal/mol for the (010) surface and from −12.35 to 0.15 kcal/mol for the (100) surface (Figure 12c). The relatively less negative distributions indicate weaker adsorption and lower overall interaction strength between the chitin and Ag/Ni(OH)_2_ surface. Accordingly, the calculated interaction with chitin should be considered a computational prediction of potential interactions with a fungal cell-wall component rather than experimental evidence of antifungal activity. Therefore, the energy distribution profiles are consistent with the general adsorption–strength sequence of LPS > ergosterol > chitin obtained from the calculated adsorption energies. The predominance of more negative energy configurations for LPS indicates a greater energetic propensity for adsorption on Ag/Ni(OH)_2_ surfaces, supporting its stronger interaction with bacterial outer-membrane components. The (100) surface also shows a broader extension toward negative energies for LPS and ergosterol, which is consistent with their more favorable adsorption energies on this facet. However, the energy distribution profiles do not necessarily reproduce the exact ranking obtained from the average or representative adsorption energies. For example, compared with that on the (010) surface, the distribution of chitin on the (100) surface extends slightly further toward negative energies. However, the calculated overall adsorption energy is slightly more favorable for the (010) surface. This apparent difference arises because the energy distribution describes the range and population of the sampled configurations, whereas the adsorption energy represents the energetic favorability of the selected adsorbed state. Therefore, the energy distribution and adsorption energy analyses should be regarded as complementary rather than contradictory. Together, they provide information on both the diversity of energetically accessible adsorption configurations and the overall stability of the preferred adsorbed state.

The RDF profiles provide complementary structural information on the interfacial organization of LPS, ergosterol, and chitin on Ag/Ni(OH)_2_ surfaces, as shown in Figure 13. For LPS, both the (010) and (100) surfaces exhibit a pronounced first RDF peak at short intermolecular distances, within the 1.83–2.39 Å range (Figure 13a), indicating a preferential spatial arrangement of LPS near the surface. A broader feature is also observed at 3.07–3.65 Å, which may reflect additional short-range contacts involving the functional groups of LPS and surface species. At greater distances, the RDF oscillations progressively decrease, indicating that structural ordering is predominantly localized within the interfacial region. Compared with the (100) surface (g(r) = 9.87), the (010) surface has a higher first RDF peak intensity (g(r) = 12.68), indicating a more pronounced local structural ordering of LPS at the (010) interface. However, this higher RDF peak does not necessarily imply more favorable overall adsorption energy. In fact, the results of the adsorption-energy analysis revealed more favorable adsorption of LPS on the (100) surface. This apparent difference arises because the RDF describes the spatial distribution and local ordering of the adsorbate relative to the surface, whereas the adsorption energy reflects the energetic favorability of the adsorbed configuration. Thus, the higher RDF intensity on the (010) surface indicates stronger local structural organization, whereas the more negative adsorption energy on the (100) surface indicates greater energetic stabilization [94,95]. With respect to ergosterol, the RDF profiles display less pronounced and broader features, with a main feature centered at 1.91 Å and additional contributions at greater distances (Figure 13b). These features indicate a less strongly ordered interfacial structure than the highly organized adsorption configurations observed for LPS and chitin. The absence of a dominant interaction mode may involve a combination of nonspecific surface contacts and dispersion-related contributions of the steroidal structure. A weak shoulder at approximately 3.53 Å further indicates additional, less pronounced adsorption configurations. Beyond 4.21 Å, the RDF profiles show reduced structural organization, with the g(r) values decreasing to 2.88 for the (010) surface and 2.44 for the (100) surface. Compared with the (100) surface, the (010) surface has a somewhat higher RDF peak intensity, indicating greater local interfacial ordering. Nevertheless, the adsorption energy results indicate slightly more favorable overall adsorption on the (100) surface. Therefore, as observed for LPS, the higher RDF intensity on the (010) surface should not be interpreted as evidence of stronger adsorption; rather, it indicates a greater degree of local spatial ordering at the interface. With respect to chitin (Figure 13c), the RDF analysis reveals a distinct first sharp peak at 1.89 Å, indicating a preferred short-range arrangement between chitin and Ag/Ni(OH)_2_ surface [96]. A broader feature of approximately 3.13 Å suggests the presence of additional short-range contacts distributed along the chitin molecule. The first RDF peak intensities reach g(r) = 14.80 for the (010) surface and 11.13 for the (100) surface, indicating more pronounced local interfacial ordering on the (010) surface. In this case, the RDF results are consistent with the results of the adsorption-energy calculations, which also indicate slightly more favorable adsorption of chitin on the (010) surface. The higher and sharper RDF features for the (010) orientation therefore suggest a more organized local arrangement of chitin at this interface [97,98].

Hence, the RDF results demonstrate that the two crystallographic surfaces can produce different degrees of local interfacial ordering. A higher g(r) value indicates a greater probability of finding the corresponding species at a particular distance and therefore reflects local structural ordering. More negative adsorption energy indicates greater overall energetic favorability of the adsorbed state. Consequently, the higher RDF intensities observed for LPS and ergosterol on the (010) surface do not contradict the more favorable adsorption energies calculated for the (100) surface. Rather, these results indicate that the (010) surface promotes greater local structural organization, whereas the (100) surface provides greater overall energetic stabilization for these two biomolecules. For chitin, both analyses consistently favor the (010) surface. Thus, the combined adsorption-energy and RDF analyses provide complementary insights into the adsorption process by describing its energetic stability and spatial organization, respectively.

## 4. Conclusions

*Klebsiella pneumoniae* is prevalent in human and animal samples and poses a serious risk to public health, especially among isolates that carry virulence markers and exhibit resistance to the majority of commonly used antibiotics, which is linked to treatment failure. Additionally, the promising antibacterial and anti-biofilm activities of Ag/Ni(OH)_2_/Clove nanocomposite, which exhibited good biocompatibility and low cytotoxicity only within the lower tested concentration range (7.8–62.5 µg/mL, cell viability > 90.0%) and showed a marked, concentration-dependent decline in cell viability at higher doses (IC50 = 264.7 µg/mL), provide evidence for improving the efficacy of available drugs when used within this favorable, lower concentration window. Furthermore, the simulation results suggest that Ag/Ni(OH)_2_ strongly attracted key microbial components, supporting its potential antimicrobial activity. Moreover, the noticeable difference between the (010) and (100) surfaces highlights the critical role of crystallographic orientation in determining the adsorption strength and surface reactivity. Monte Carlo (MC) simulations were carried out to evaluate the adsorption behavior of representative bacterial and fungal biomolecules on Ag/Ni(OH)_2_ composite surface. Lipopolysaccharide (LPS), the principal component of the outer membrane of Gram-negative bacteria, and ergosterol and chitin, which are characteristic constituents of fungal membranes and cell walls, respectively, were employed as model adsorbents. The obtained adsorption energies of −50.15, −23.46, and −8.08 kcal/mol for LPS, ergosterol, and chitin, respectively, reveal spontaneous and energetically favorable adsorption, following the order LPS > ergosterol > chitin. The strong affinity between the composite surface and these biomolecules provides a molecular-level explanation for the enhanced antibacterial activity observed in the experimental investigations.

In addition to the strong affinity between the nanocomposite surface and bacterial cells, several molecular-level mechanisms may contribute to the observed antibacterial activity. In particular, Ag-containing nanomaterials can exert antibacterial effects through the release of Ag+ ions, interactions with thiol-containing proteins and enzymes, disruption of cellular membrane integrity, and induction of oxidative stress. Ni(OH)_2_ component may additionally contribute through surface interactions with bacterial cell envelopes and interference with essential cellular processes. Furthermore, the nanoscale structure of the composite can facilitate close contact with bacterial cells, thereby enhancing these interactions.

## Figures and Tables

**Figure 1:**
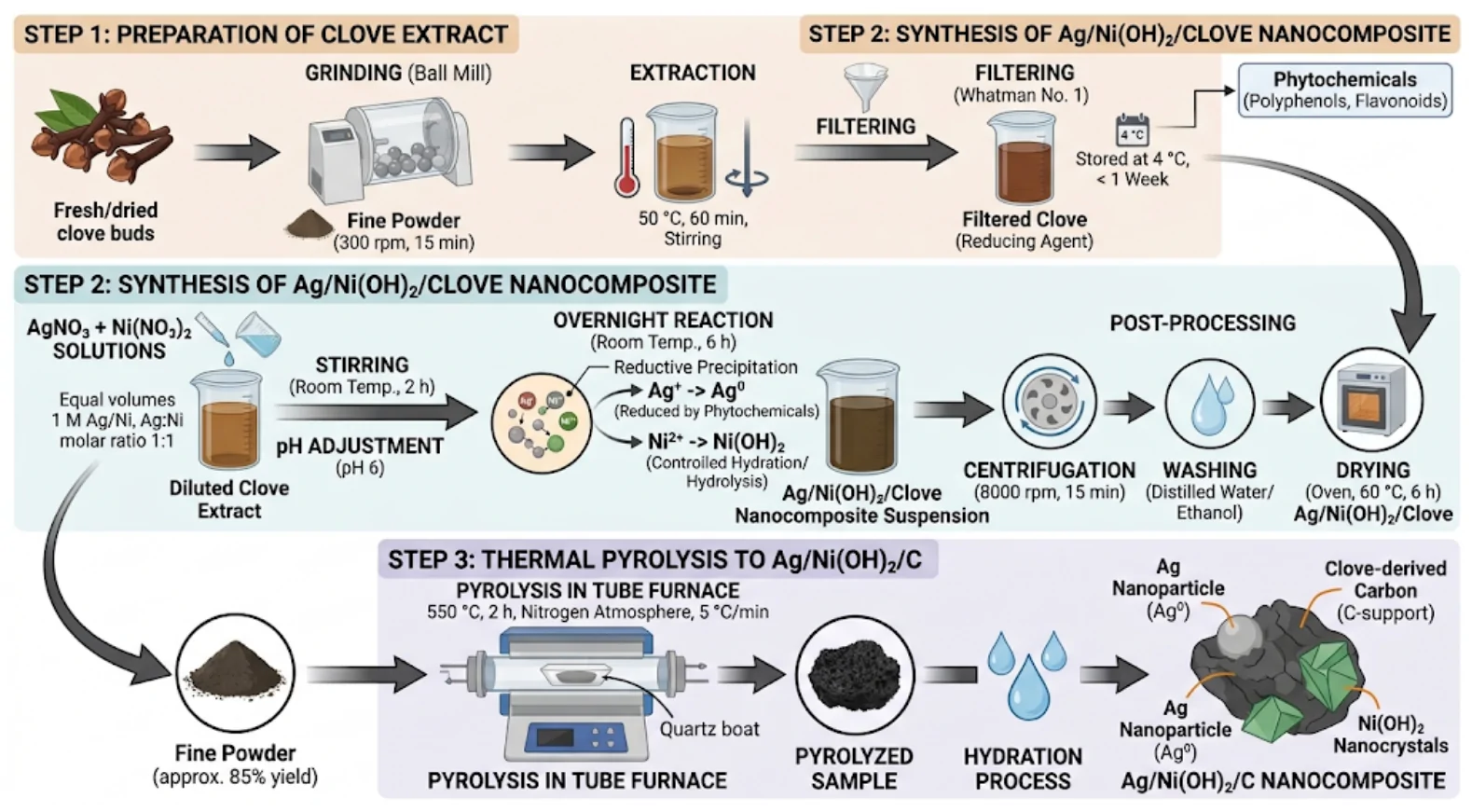
Preparation steps of the extract, Ag/Ni(OH)_2_/Clove and Ag/Ni(OH)_2_/C nanocomposites.

**Figure 2:**
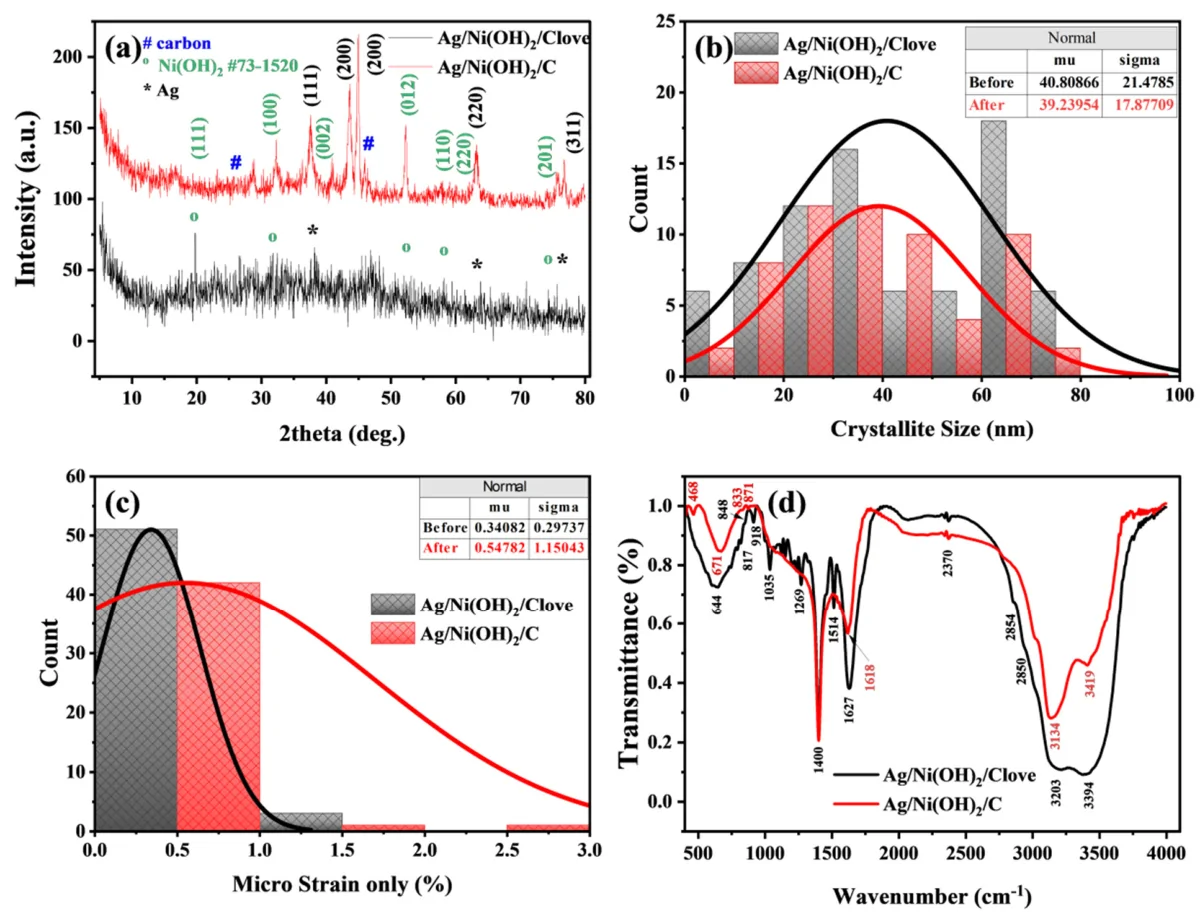
(**a**) XRD analysis, (**b**) crystallite size distribution, (**c**) microstrain distribution, and (**d**) FTIR spectra of the Ag/Ni(OH)_2_/Clove nanocomposite and Ag/Ni(OH)_2_/C.

**Figure 3:**
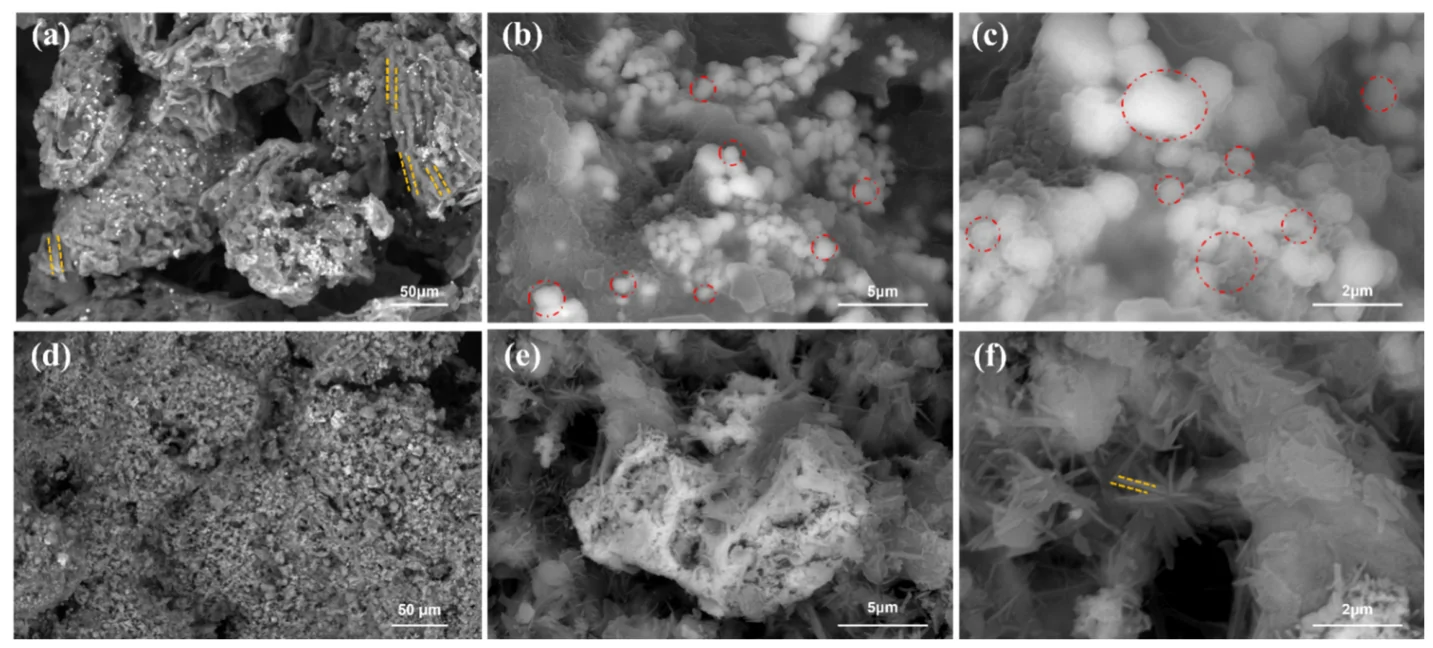
FE-SEM images of the Ag/Ni(OH)_2_/Clove nanocomposite at (**a**) 1000×, (**b**) 16,000×, and (**c**) 40,000× magnification. FE-SEM images of the Ag/Ni(OH)_2_/C nanocomposite at (**d**) 1000×, (**e**) 16,000×, and (**f**) 40,000× magnification. (yellow line is wrinkle and red cycle is particle).

**Figure 4:**
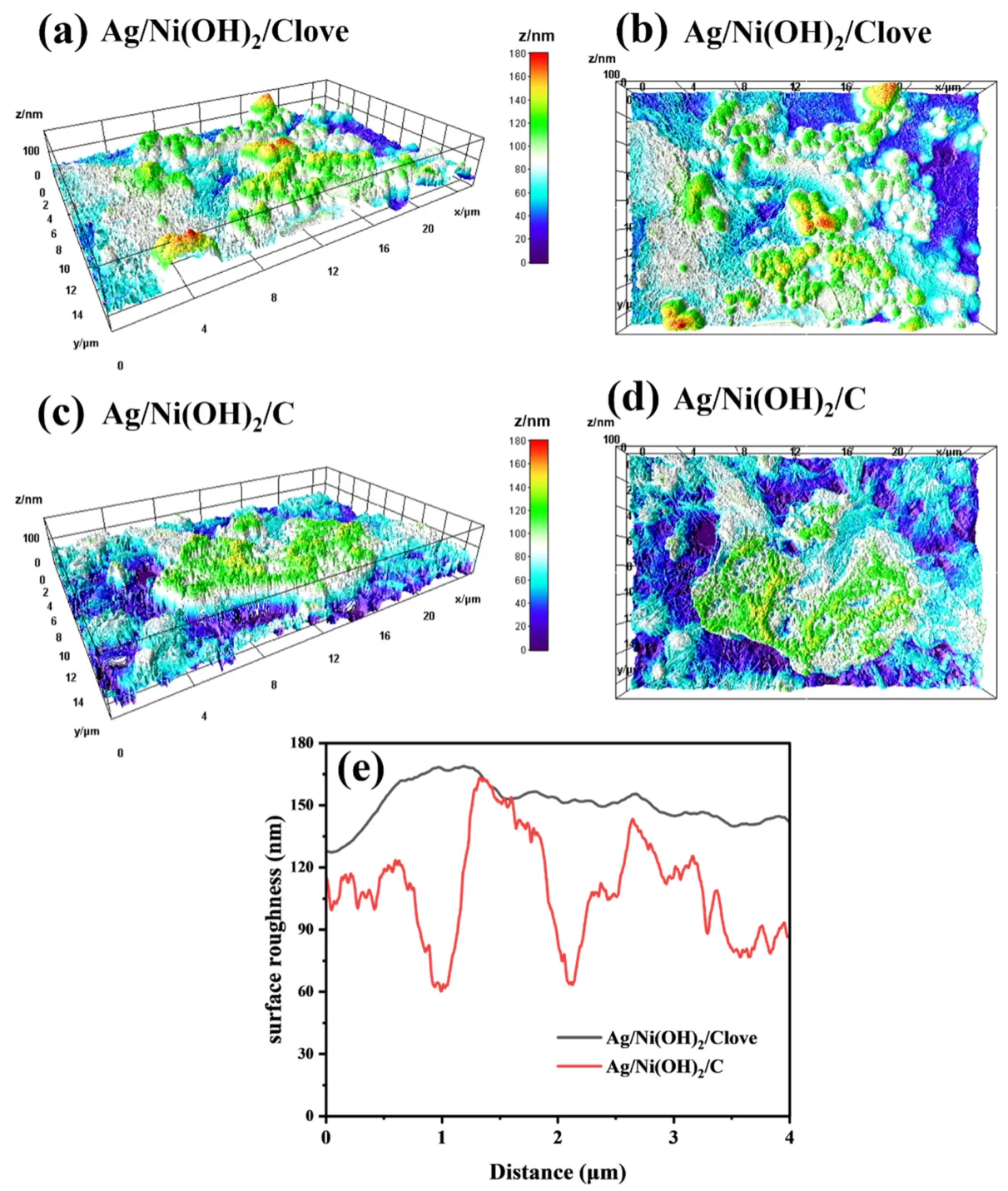
Side and top views of 3D topography for (**a**,**b**) Ag/Ni(OH)_2_/Clove and (**c**,**d**) Ag/Ni(OH)_2_/C nanocomposites. (**e**) Surface roughness analysis.

**Figure 5:**
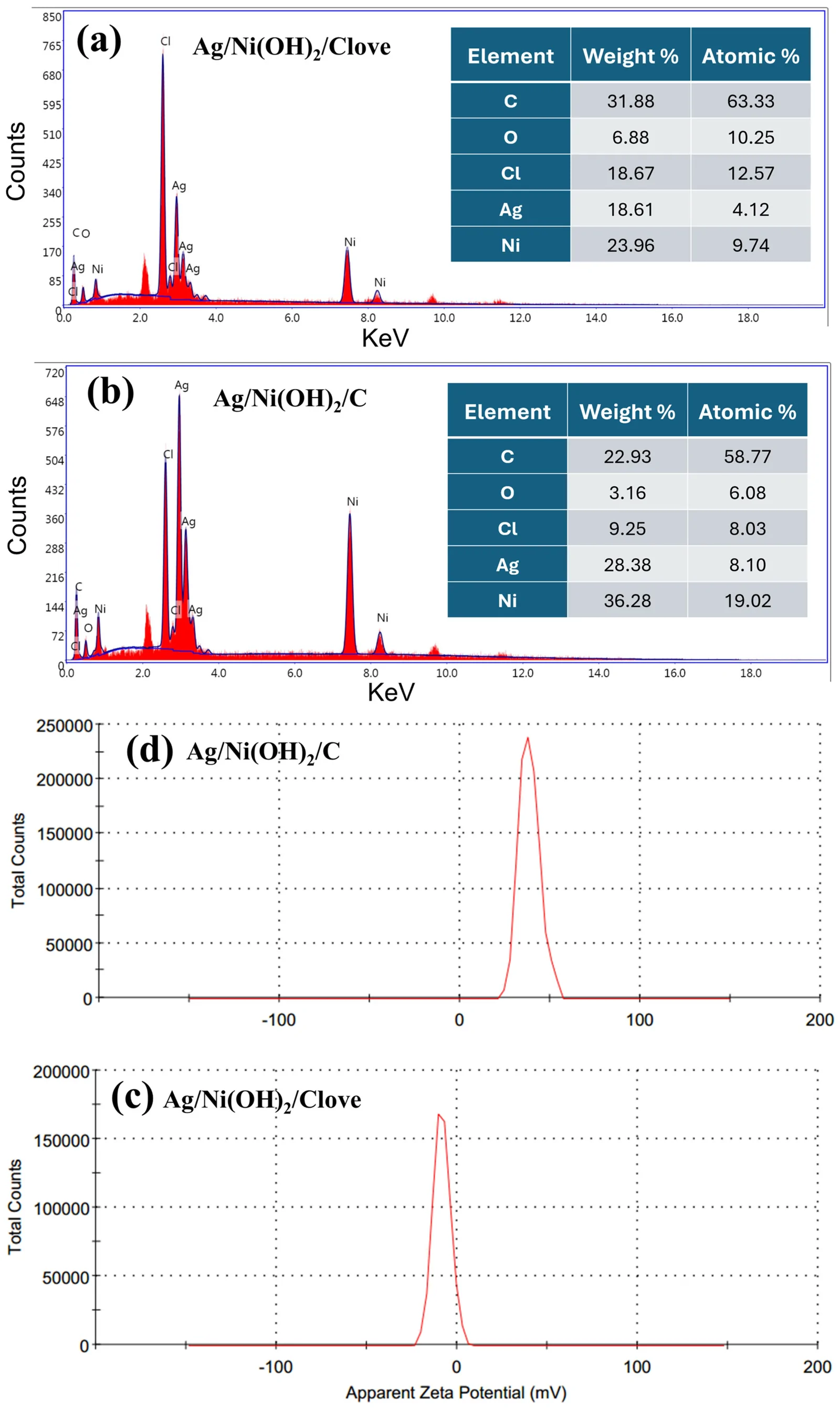
EDX spectra of the (**a**) Ag/Ni(OH)_2_/Clove and (**b**) Ag/Ni(OH)_2_/C nanocomposites. Zeta potential of the (**c**) Ag/Ni(OH)_2_/Clove and (**d**) Ag/Ni(OH)_2_/C nanocomposites.

**Figure 6:**
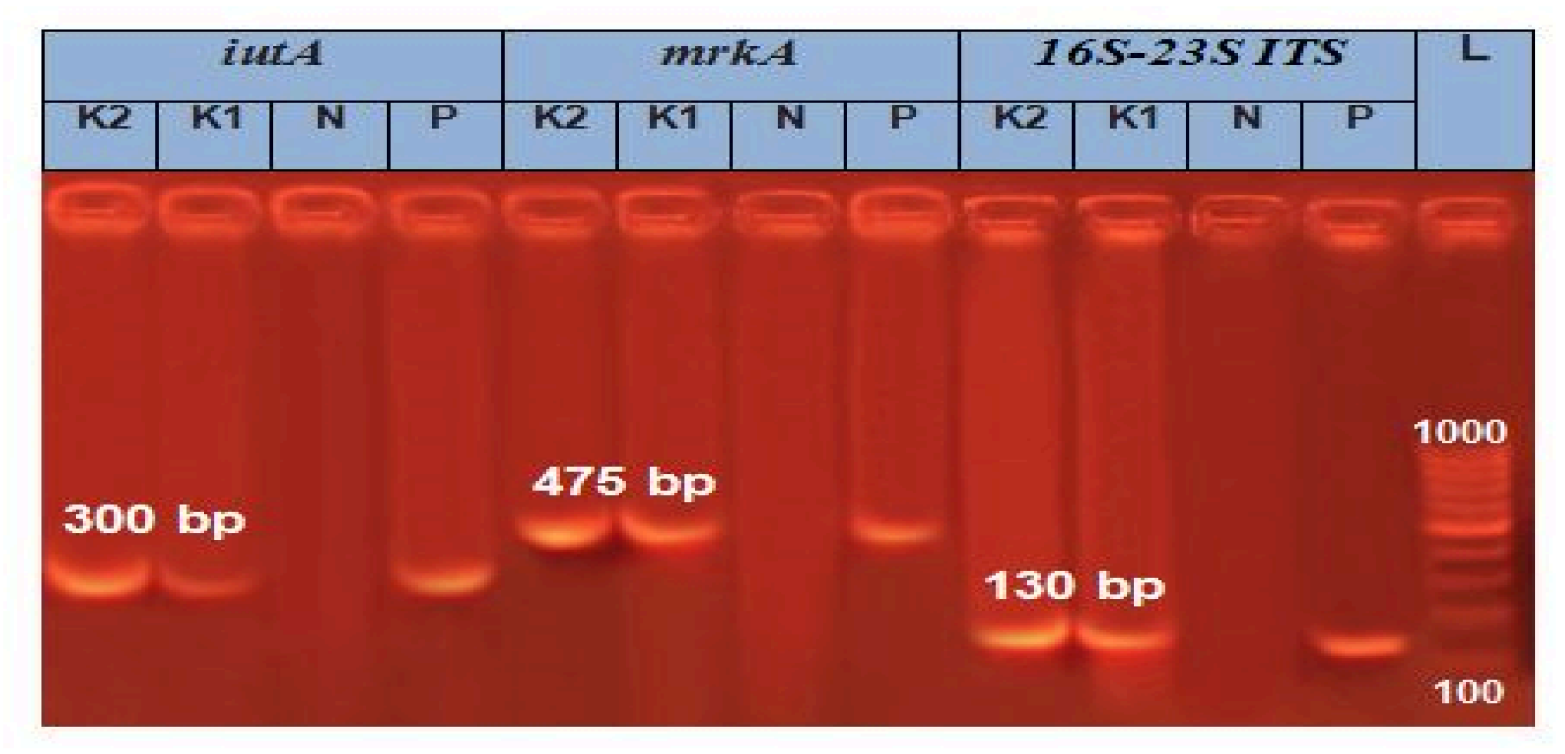
Agarose gel electrophoresis of PCR products from *K. pneumoniae* isolates showing amplification of *iut*-A gene (300 bp), *mrk*-A (475 bp), and *10S–23 ITS* (130 bp); Lane L: 100 bp DNA ladder; Lanes K1 and K2: the tested isolates; Lane P: positive control; Lane N: negative control.

**Figure 7:**
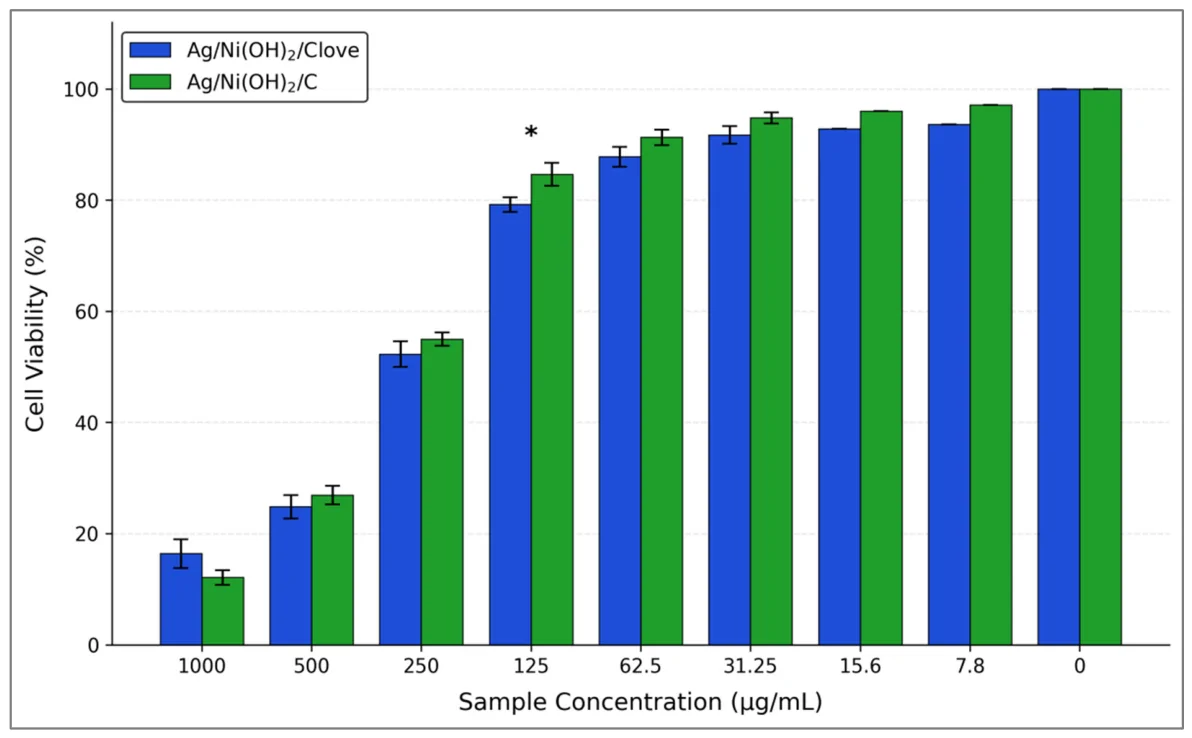
Cell viability (%) of HEK293 cells after 24 h of exposure (MTT assay, *n* = 3, mean ± SD) to varying doses of Ag/Ni(OH)_2_/Clove and Ag/Ni(OH)_2_/C nanocomposites. The error bars represent the SDs; * indicates *P* < 0.05 (Welch’s *t* test) between the two formulations at that concentration.

**Figure 8:**
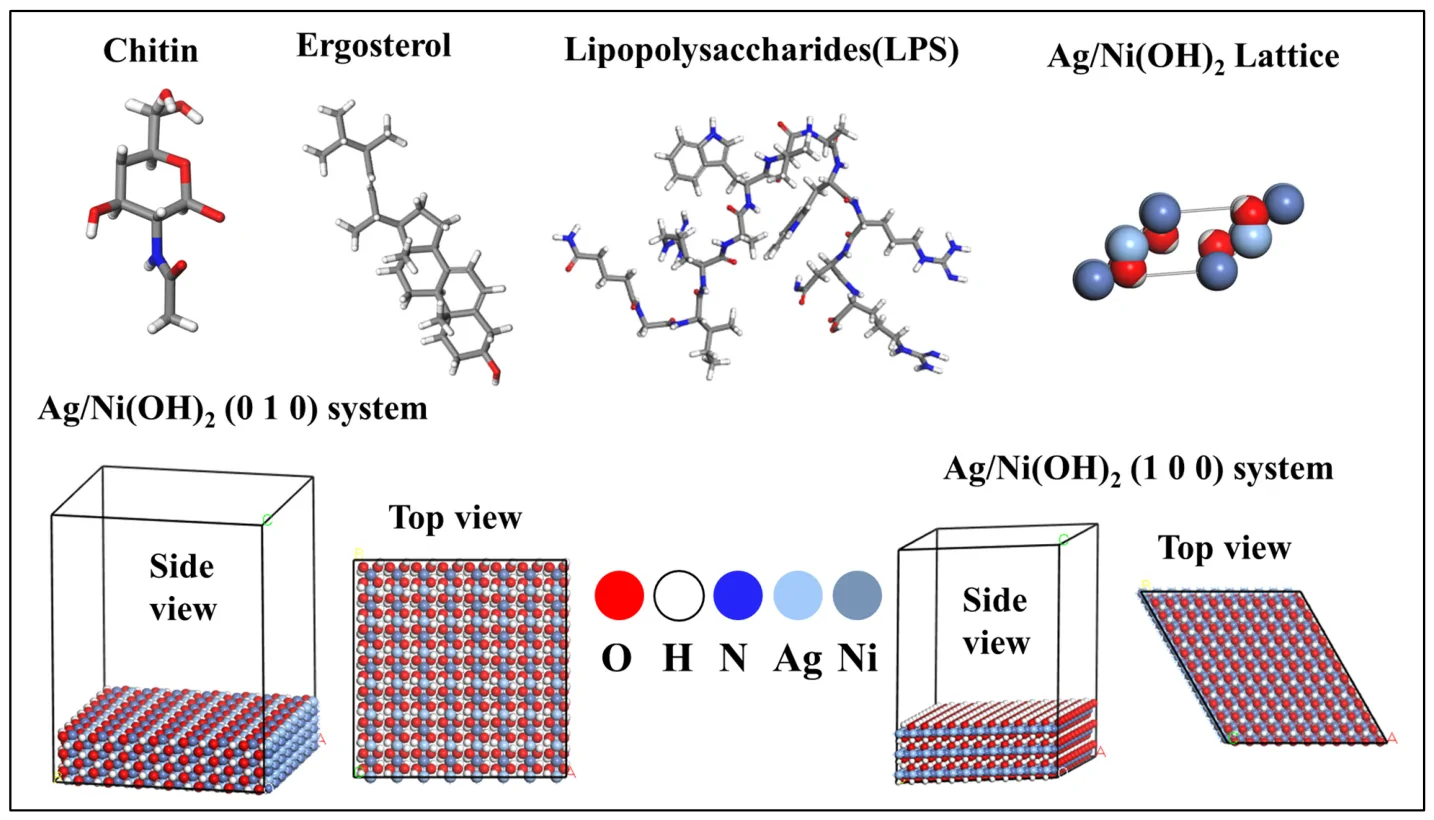
Optimized structures of the Ag/Ni(OH)_2_ lattice and surfaces with different crystallographic orientations (010) and (100), along with the biological target molecules lipopolysaccharide (LPS), ergosterol, and chitin used in the adsorption study.

**Figure 9:**
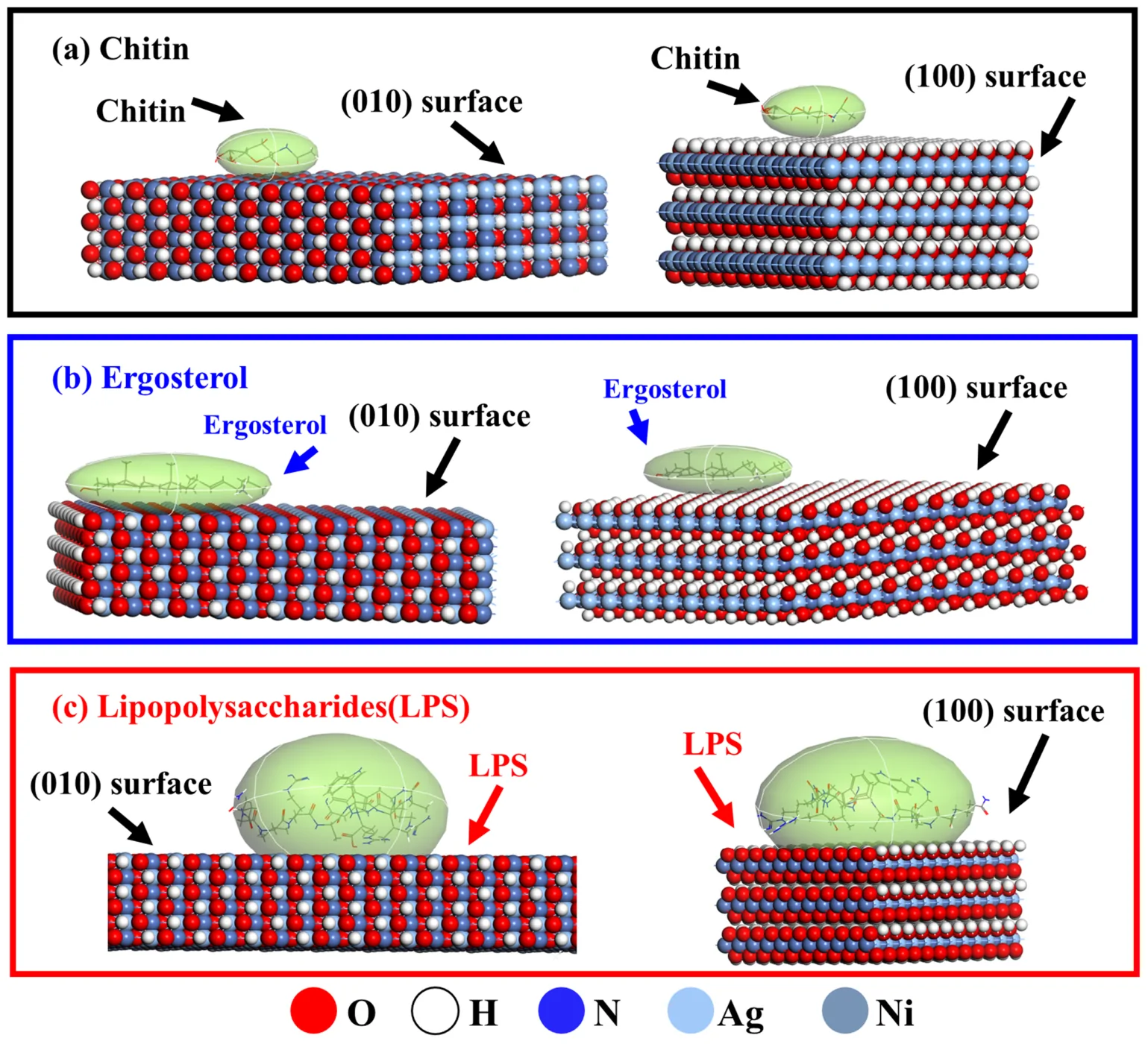
Equilibrium adsorption configurations of (**a**) LPS, (**b**) ergosterol, and (**c**) chitin on Ag/Ni(OH)_2_ (010) and (100) surfaces.

**Figure 10:**
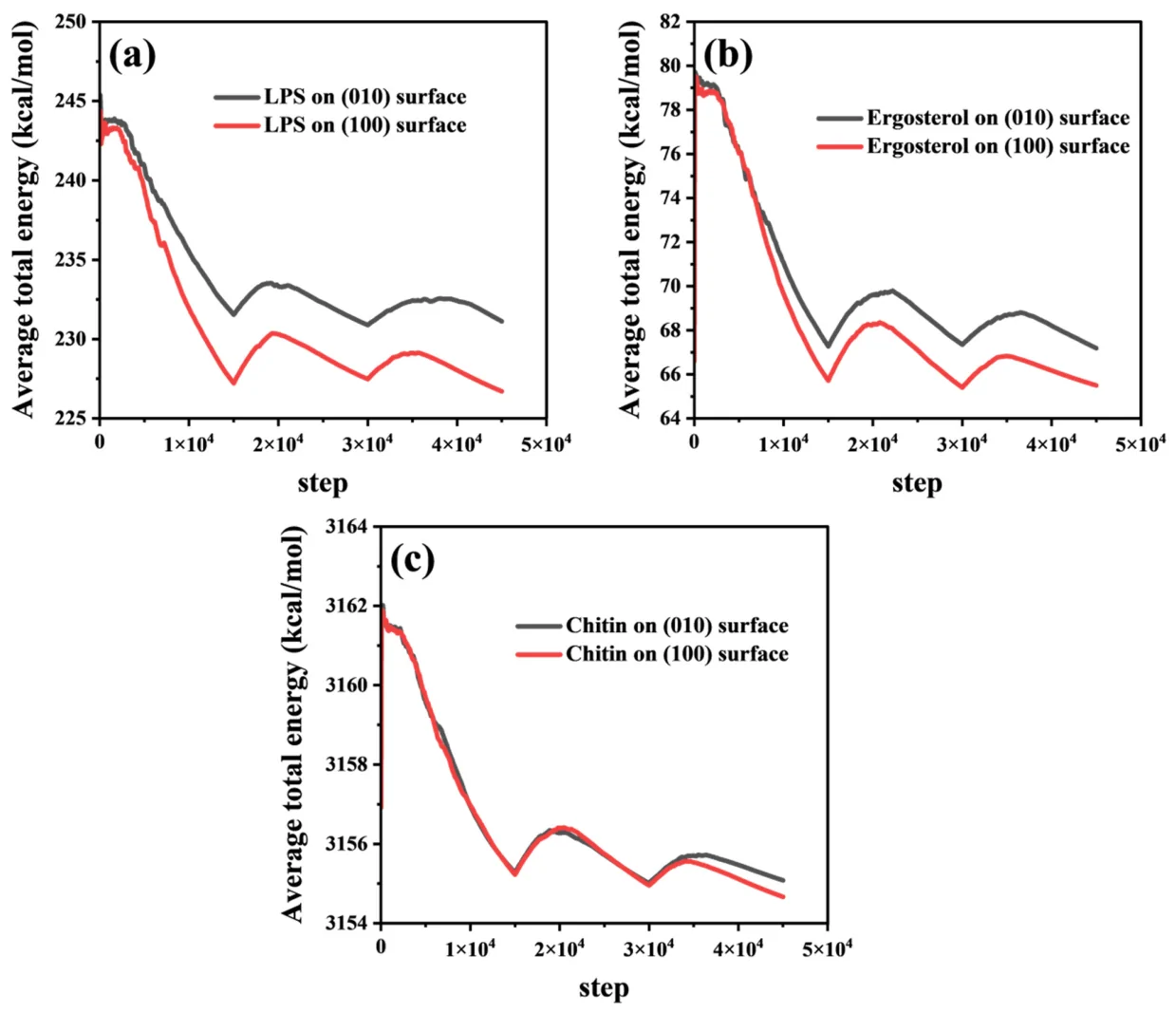
Average total energy profiles obtained during the Monte Carlo simulations for (**a**) LPS, (**b**) ergosterol, and (**c**) chitin on the Ag/Ni(OH)_2_(010) and (100) surfaces.

**Figure 11:**
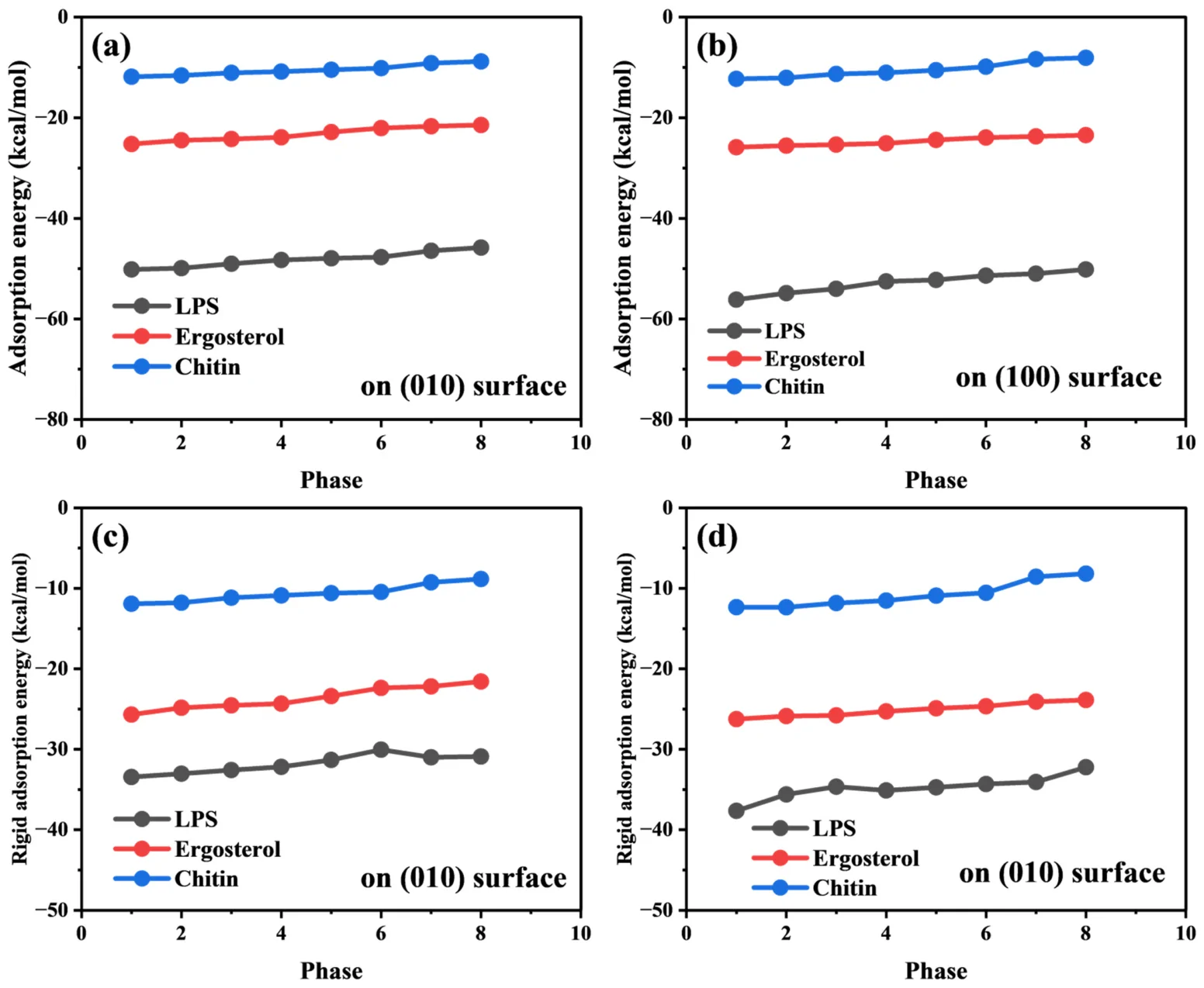
Adsorption energy profiles on (**a**) (010) surfaces and (**b**) (100) surface orientation of Ag/Ni(OH)_2_. Rigid adsorption energy profiles on (**c**) (010) surfaces and (**d**) (100) surface orientation of Ag/Ni(OH)_2_ as a function of the number of Monte Carlo simulations.

**Figure 12:**
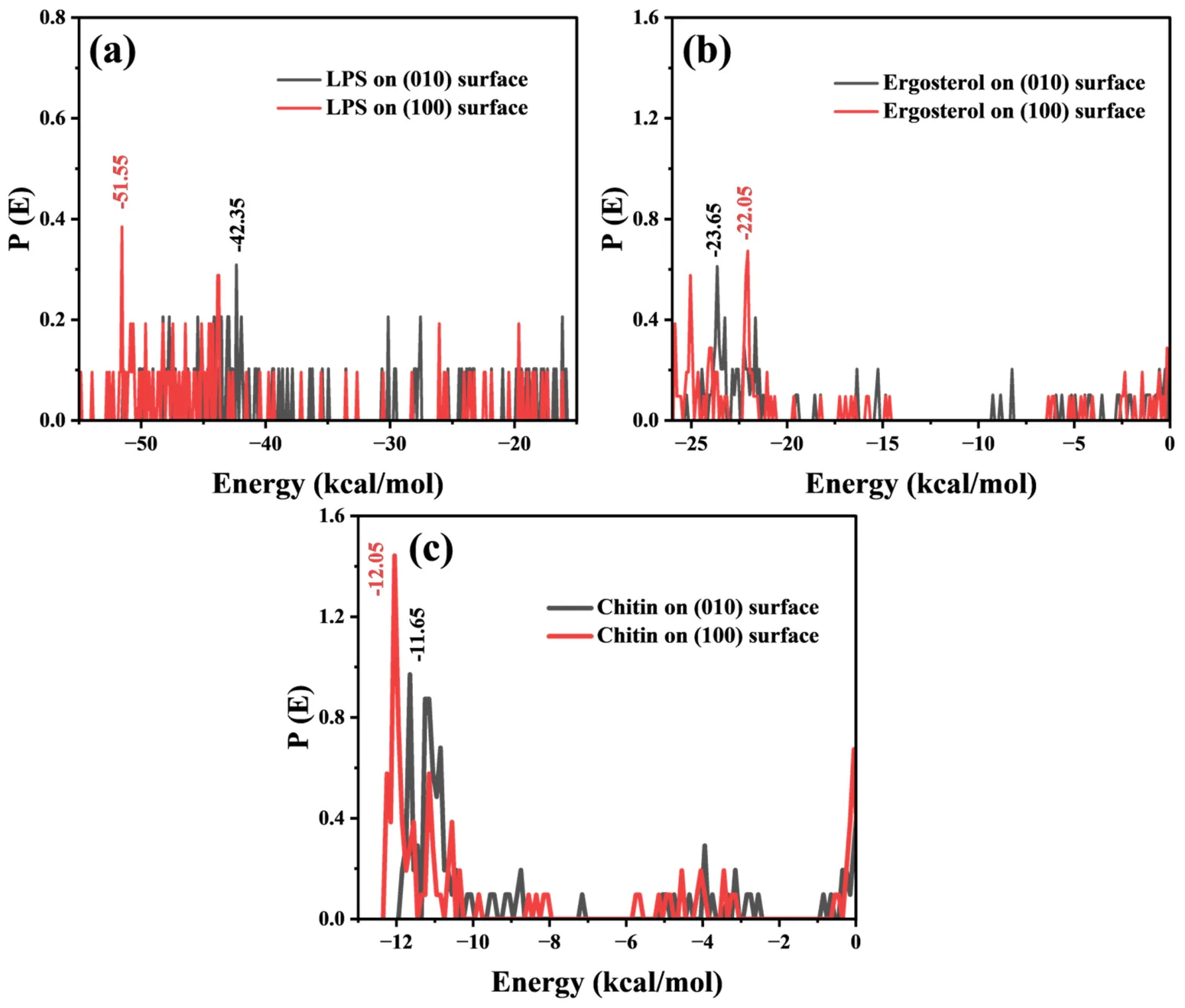
Probability distributions of the adsorption energies obtained from the Monte Carlo simulations for (**a**) LPS, (**b**) ergosterol, and (**c**) chitin on the Ag/Ni(OH)_2_ (010) and (100) surfaces (black and red curves correspond to the (010) and (100) surface orientations, respectively).

**Figure 13:**
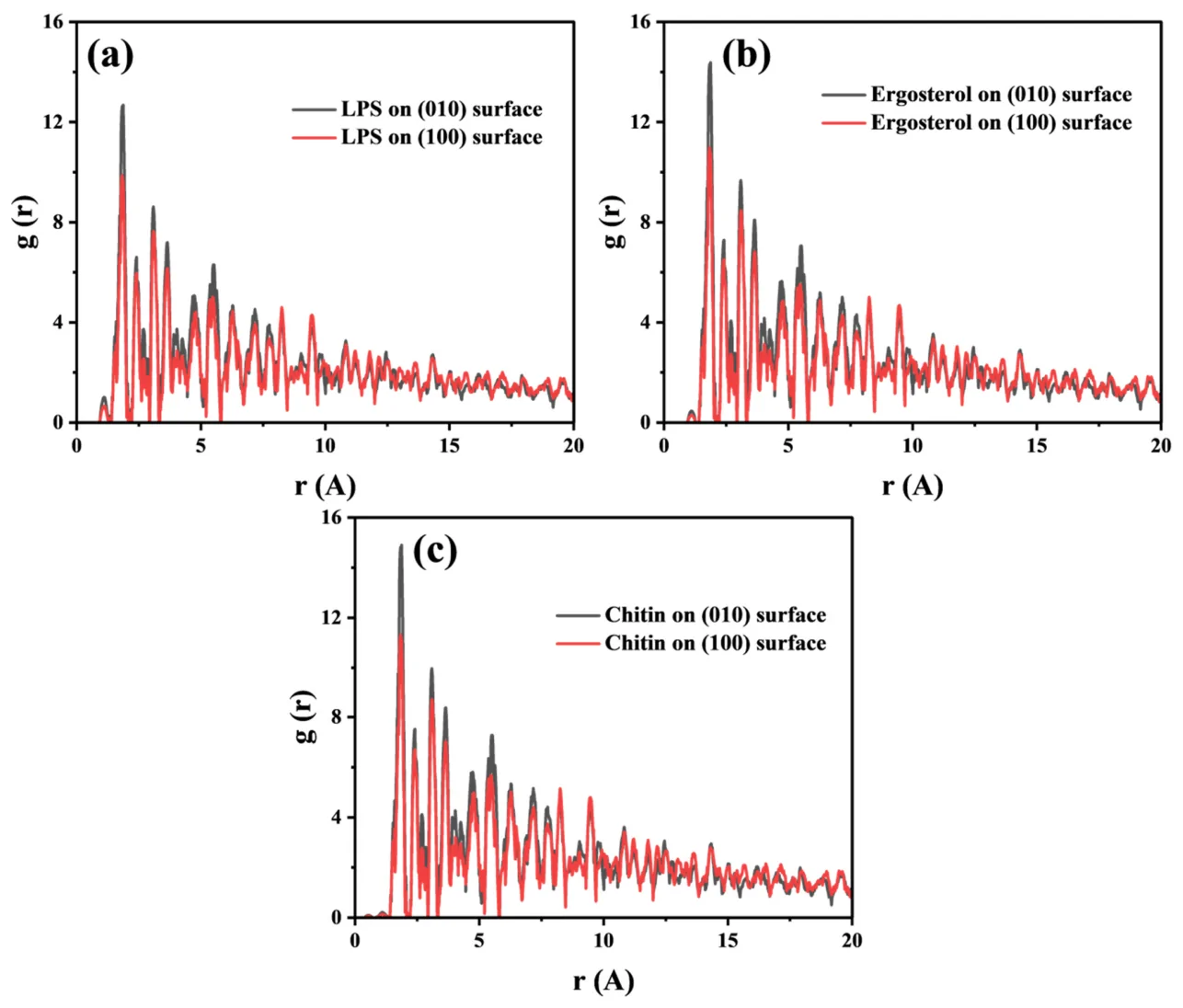
RDFs describing the spatial distribution of (**a**) LPS, (**b**) ergosterol, and (**c**) chitin relative to the Ag/Ni(OH)_2_ surfaces for the (010) and (100) surfaces.

**Table 1:** Primer sequences, target genes, amplicon sizes and annealing temperatures specific for *K. pneumoniae* in the current study.

Target Genes	Primers Sequences	Amplified Segment (bp)	Annealing	References
*Mrk*-A	CGGTAAAGTTACCGACGTATCTTGTACTG	475	55 °C	[16]
	GCTGTTAACCACACCGGTGGTAAC			
*16S–23S ITS*	ATTTGAAGAGGTTGCAAACGAT	130	55 °C	[17]
	TTCACTCTGAAGTTTTCTTGTGTTC			
*Iut*-A	GGCTGGACATGGGAACTGG	300	63 °C	[18]
	CGTCGGGAACGGGTAGAATCG			

**Table 2:** Prevalence of *K. pneumoniae* in the examined cattle and humans.

Samples	Number Examined	Number Positive (%)
Feces	50	24 (48.0)
Milk	50	19 (38.0)
Stool	30	13 (43.3)
Total	130	56 (43.1)
*P*-value	1.021, X_2_ = 0.6003	

**Table 3:** Antimicrobial resistance pattern of *K. pneumoniae* in the examined animal and human samples.

Sample/Number Examined	Antibiotics Used (Number Positive (%))																					
	Amoxicillin/Clavulanic Acid (30 µg)			SulphaTrimethoprim (SXT, 25 μg)			Fosfomycin (FOS, 20 μg)			Ciprofloxacin (CIP, 5 µg)			Amikacin (AK, 30 μg)			Gentamicin (GEN, 10 µg)			Doxycycline (DO, 30 μg)			*P*-Value
	S	I	R	S	I	R	S	I	R	S	I	R	S	I	R	S	I	R	S	I	R	
Feces (24)	3 (12.5)	2 (8.3)	19 (79.2)	3 (12.5)	3 (12.5)	18 (75.0)	6 (25.0)	3 (12.5)	15 (62.5)	5 (20.8)	- (0.0)	19 (79.2)	7 (29.2)	5 (20.8)	12 (50.0)	5 (20.8)	5 (20.8)	14 (58.3)	3 (12.5)	3 (12.5)	18 (75.0)	0.428, X_2_= 12.220
Milk (19)	1 (5.3)	3 (15.8)	15 (78.9)	3 (15.8)	2 (10.5)	14 (73.7)	4 (21.1)	2 (10.5)	13 (68.4)	5 (26.3)	3 (15.8)	11 (57.9)	4 (21.0)	2 (10.5)	13 (68.4)	2 (10.5)	3 (15.8)	14 (73.7)	2 (10.5)	3 (15.8)	14 (73.7)	0.944, X2= 5.390
Stool (13)	2 (15.4)	2 (15.4)	9 (69.2)	1 (7.7)	2 (15.4)	10 (76.9)	3 (23.1)	3 (23.1)	7 (53.8)	4 (30.8)	2 (15.4)	7 (53.8)	3 (23.1)	- (0.0)	10 (76.9)	2 (15.4)	1 (7.7)	10 (76.9)	4 (30.8)	- (0.0)	9 (69.2)	0.672, X_2_ = 9.363
*P*-value	0.814, X_2_ = 1.537			0.967, X_2_ = 0.561			0.865, X_2_ = 1.279			0.266, X_2_ = 5.214			0.347, X_2_ = 4.461			0.7015, X_2_ = 2.187			0.372, X_2_ = 4.262			

S, Sensitive; I, Intermediate; R, Resistant.

**Table 4:** Biofilm formation grades of *K. pneumoniae* isolates from the examined cattle and humans.

Samples/Number Examined	Biofilm Formation Results (Number Examined/Percentage (%))			Non Biofilm Producers	*P*-Value
	Biofilm Formation Grades				
	Strong (+++)	Moderate (++)	Weak (+)		
Feces (*n* = 24)	9 (37.5)	6 (25.0)	4 (16.7)	5 (20.8)	0.5062
Milk (*n* = 19)	7 (36.8)	5 (26.3)	3 (15.8)	4 (21.1)	0.6058
Stool (*n* = 13)	6 (46.2)	4 (30.8)	1(7.7)	2 (15.3)	0.2089
Total (*n*= 56)	22 (39.3)	15 (26.8)	8 (14.3)	11 (19.6)	
*P*-value			0.986, X_2_ = 0.989		

“*n*”: the number of *K. pneumoniae* isolates per sample type.

**Table 5:** Antimicrobial and antibiofilm activity of Ag/Ni(OH)_2_/Clove and Ag/Ni(OH)_2_/C nanocomposite formulas against *K. pneumoniae* in animals and humans.

Antimicrobial Patterns	Ag/Ni(OH)_2_/Clove Nanocomposite	Ag/Ni(OH)_2_/C Nanocomposite
Inhibition zone (mm)	14.00 ± 1.15	20.67 ± 1.76 *
MIC (μg/mL)	48.77 ± 9.20	20.83 ± 5.23
MBC (μg/mL)	104.17 ± 20.83	36.50 ± 13.81
MBC/MIC Ratio	2.1	1.7
	**Biofilm inhibition percentages (%)**	
Concentration (μg/mL)		
100	3 (20.0)	5 (33.3)
500	6 (40.0)	8 (53.3)
750	9 (60.0)	11 (73.3)
1000	10 (66.7)	13 (86.7)
*P*-value	0.0453, X_2_ = 28.036	0.0157, X_2_ = 10.364

The data are expressed as the means ± SEs; * indicates a significant difference at *P* ˂ 0.05.

**Table 6:** MTT cell viability data for HEK293 cells exposed to Ag/Ni(OH)_2_/Clove and Ag/Ni(OH)_2_/C nanocomposites for 24 h (*n* = 3, mean ± SD).

Concentration (µg/mL)	Ag/Ni (OH)_2_/Clove (%)	Ag/Ni (OH)_2_/C (%)	*P*-Value (Welch’s *t* Test)
1000	16.38 ± 2.55	12.09 ± 1.32	0.081
500	24.81 ± 2.10	26.91 ± 1.70	0.252
250	52.27 ± 2.30	55.00 ± 1.20	0.166
125	79.18 ± 1.30	84.62 ± 2.10	0.026 *
62.5	87.77 ± 1.80	91.28 ± 1.40	0.060
31.25	91.67 ± 1.60	94.79 ± 1.00	0.056
15.6	92.84	95.96	—
7.8	93.62	97.13	—
0	100.00	100.00	—

* *P* < 0.05. IC50 (log-linear interpolation): 264.7 µg/mL for Ag/Ni(OH)_2_/Clove and 282.8 µg/mL for Ag/Ni(OH)_2_/C.

## Data Availability

The datasets generated and/or analyzed during this study are available from the corresponding author upon reasonable request.
